# *Tbx21* and *Foxp3* Are Epigenetically Stabilized in T-Bet^+^ Tregs That Transiently Accumulate in Influenza A Virus-Infected Lungs

**DOI:** 10.3390/ijms22147522

**Published:** 2021-07-14

**Authors:** Yassin Elfaki, Juhao Yang, Julia Boehme, Kristin Schultz, Dunja Bruder, Christine S. Falk, Jochen Huehn, Stefan Floess

**Affiliations:** 1 Department of Experimental Immunology, Helmholtz Centre for Infection Research, 38124 Braunschweig, Germany; y.elfaki@ucl.ac.uk (Y.E.); chypeter001@aliyun.com (J.Y.); 2 Immune Regulation Group, Helmholtz Centre for Infection Research, 38124 Braunschweig, Germany; julia.boehme@helmholtz-hzi.de (J.B.); kristin.schultz@helmholtz-hzi.de (K.S.); dunja.bruder@med.ovgu.de (D.B.); 3 Infection Immunology Group, Institute of Medical Microbiology, Infection Control and Prevention, Health Campus Immunology, Infectiology and Inflammation, Otto-von-Guericke University Magdeburg, 39120 Magdeburg, Germany; 4 Institute of Transplant Immunology, Hannover Medical School, 30625 Hannover, Germany; falk.christine@mh-hannover.de; 5 German Center for Infection Research DZIF, Thematical Translation Unit-Immunocompromized Host (TTU-IICH), Hannover-Braunschweig Site, 30625 Hannover, Germany; 6 Cluster of Excellence RESIST (EXC 2155), Hannover Medical School, 30625 Hannover, Germany

**Keywords:** influenza A virus, Tregs, lung, inflammation, methylation, *Tbx21*, *Foxp3*

## Abstract

During influenza A virus (IAV) infections, CD4^+^ T cell responses within infected lungs mainly involve T helper 1 (Th1) and regulatory T cells (Tregs). Th1-mediated responses favor the co-expression of T-box transcription factor 21 (T-bet) in Foxp3^+^ Tregs, enabling the efficient Treg control of Th1 responses in infected tissues. So far, the exact accumulation kinetics of T cell subsets in the lungs and lung-draining lymph nodes (dLN) of IAV-infected mice is incompletely understood, and the epigenetic signature of Tregs accumulating in infected lungs has not been investigated. Here, we report that the total T cell and the two-step Treg accumulation in IAV-infected lungs is transient, whereas the change in the ratio of CD4^+^ to CD8^+^ T cells is more durable. Within lungs, the frequency of Tregs co-expressing T-bet is steadily, yet transiently, increasing with a peak at Day 7 post-infection. Interestingly, T-bet^+^ Tregs accumulating in IAV-infected lungs displayed a strongly demethylated *Tbx21* locus, similarly as in T-bet^+^ conventional T cells, and a fully demethylated Treg-specific demethylated region (TSDR) within the *Foxp3* locus. In summary, our data suggest that T-bet^+^ but not T-bet^−^ Tregs are epigenetically stabilized during IAV-induced infection in the lung.

## 1. Introduction

Regulatory T cells (Tregs) constitute a CD4^+^ T cell population that controls other immune cells to limit immune reactions and guarantee tolerance towards the body’s own cells, food or symbiotic microorganisms. A loss of Tregs leads to severe autoimmune phenotypes such as scurfy in mice or IPEX in humans [1,2,3,4]. Foxp3 was identified as a lineage-specification factor being essential for the functional properties of Tregs [5]. As a part of the T cell compartment, the majority of the Foxp3^+^ Treg population originates from the thymus [6]. In addition, a substantial proportion of Tregs develops in the periphery via conversion of naïve CD4^+^ T cells into Foxp3^+^ Tregs under tolerogenic conditions to prevent immune reactions against harmless antigens such as commensal and food-borne antigens [7].

The stable and continuous gene expression of Foxp3, which is critical for the maintenance of the functional properties of Tregs, is enabled by DNA demethylation of an evolutionarily conserved region named Treg-specific demethylated region (TSDR), located in the first intron of the *Foxp3* gene locus [8]. Subsequent studies revealed that an active, incremental demethylation process during thymic Treg development is involved, starting upon Foxp3 induction, continued during further differentiation and completed after egress into peripheral tissues [9,10]. It is important to note that regulation of gene expression by epigenetic mechanisms is a common feature of T cell development, demonstrated by changes of the methylation status in key gene loci such as *Tbx21*, *Ifng*, *Il4* or *Il17a* contributing to the functional phenotype of differentiated CD4^+^ T helper (Th) 1, 2 or 17 subsets [11].

A new era in Treg research began when it was discovered that Foxp3^+^ Tregs can express Th cell-specific transcription factors such as T-box transcription factor 21 (T-bet), GATA binding protein 3 (GATA3) or retinoic acid-related orphan receptor (ROR)γt [12,13,14,15], demonstrating a high degree of heterogeneity and functional specialization within the Treg population and providing strong evidence that Th cell-like transcriptional programs in Tregs constitute a general mechanism for the specific control of CD4^+^ T cell-mediated immune reactions [16]. T-bet^+^Foxp3^+^ Tregs were reported to be induced upon various infections and to efficiently control Th1-mediated (auto) immune responses [13,17,18]. However, some aspects of the functionality of T-bet^+^ Tregs still remain unclear, as one report described the suppression of CD8^+^ T cells in a *Listeria monocytogenes*-driven model [17], whereas another recent study observed an increase in CD8^+^ tissue-resident memory T cells (T_RM_) mediated by T-bet^+^ Tregs, thereby eliciting the clearance of pathogens during *Eimeria vermiformis* and *Yersinia pseudotuberculosis* infections [19].

The CD4^+^ T cell response towards influenza A virus (IAV) infections not only involves T follicular helper (Tfh) cells, which promote germinal center formation and antibody production by B cells [20,21], but also is Th1-driven and includes the promotion of cytotoxic responses [22]. Furthermore, the accumulation of Tregs in the lung, airways and lung-draining lymph nodes (dLN) upon IAV infection was reported, with its peak preceding that of effector T cell accumulation [23]. In addition, adoptively transferred TCR transgenic Tregs were shown to upregulate T-bet upon IAV infection [24]. However, the precise kinetics of the accumulation of Treg subsets, particularly T-bet-expressing cells, in IAV-infected lungs has not been elucidated, and it is unknown whether the expression of the lineage-specifying transcription factors is stabilized by epigenetic DNA mechanisms.

In the present study, we used an IAV infection model to describe the accumulation dynamics of major T cell subsets within the lung and dLN during the course of the infection. Particular emphasis was laid on the identification and epigenetic characterization of Treg subsets within the infected lungs. While we observed a more durable alteration of the ratio of CD4^+^ to CD8^+^ T cells within the lungs post-IAV infection resulting from an increased and long-lasting accumulation of CD8^+^ T cells, the accumulation of T-bet^+^ Tregs was only transient. Notably, T-bet^+^ Tregs isolated from the lungs of IAV-infected mice revealed a strongly demethylated *Tbx21* locus and a more pronounced TSDR demethylation when compared to their T-bet^−^ counterparts, indicating that the T-bet^+^ Tregs transiently accumulating in IAV-infected lungs are epigenetically stabilized in their lineage-specific transcription factor gene loci.

## 2. Results

### 2.1. Durable Change in the Ratio of CD4^+^ to CD8^+^ T Cells in Lungs of IAV-Infected Mice

**Figure 1:**
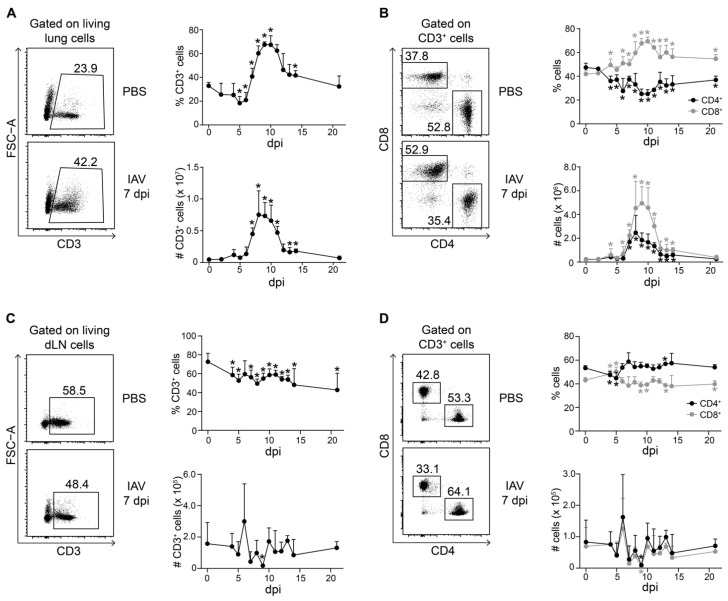
Kinetics of T cell accumulation during IAV infection. Foxp3^hCD2^ × Rag1^GFP^ reporter mice were infected with IAV and analyzed at indicated dpi. Lungs and dLNs were collected and prepared, and single-cell suspensions were restimulated and subsequently analyzed by flow cytometry. (**A**) Representative dot plots depict frequency of CD3^+^ T cells in lungs of PBS-treated and IAV-infected mice (7 dpi), gated on living lung lymphocytes. The graphs summarize frequencies (top) and absolute numbers (bottom) of CD3^+^ T cells; (**B**) Representative dot plots depict frequency of CD4^+^ and CD8^+^ T cells in lungs of PBS-treated and IAV-infected mice (7 dpi), gated on CD3^+^ T cells. The graphs summarize frequencies (top) and absolute numbers (bottom) of CD4^+^ (black filled circles) and CD8^+^ T cells (gray filled circles); (**C**) Representative dot plots depict frequency of CD3+ T cells in dLNs of PBS-treated and IAV-infected mice (7 dpi), gated on living dLN lymphocytes. The graphs summarize frequencies (top) and absolute numbers (bottom) of CD3^+^ T cells; (**D**) Representative dot plots depict frequency of CD4^+^ and CD8^+^ T cells in dLNs of PBS-treated and IAV-infected mice (7 dpi), gated on CD3^+^ T cells. The graphs summarize frequencies (top) and absolute numbers (bottom) of CD4^+^ (black filled circles) and CD8^+^ T cells (gray filled circles). Data were pooled from two to three independent experiments, which included 4–10 mice per group, and presented as mean + SD. Mann–Whitney test was used to calculate statistical significance by comparing values from untreated animals (for lung samples) or PBS-treated animals (for dLN samples) (0 dpi) with values from IAV-infected mice (2 to 21 dpi). Significant changes are indicated by an asterisk.

IAV infections pass through different phases of virus spreading in the host, accompanied by different stages of immune responses [22]. To avoid any loss of information on the T cell population dynamics during the course of the IAV infection, we performed in-depth monitoring. Female mice of the same age were infected intranasally with a mouse-adapted strain of IAV (PR8M), and the dynamics of major T cell populations within lungs and dLNs were analyzed on Day 2, daily between four and fourteen days as well as 21 days post-infection (dpi) by flow cytometry. In accordance with previously published data [25], we observed a significant increase in CD3^+^ T cells in IAV-infected lungs starting at 7 dpi and exhibiting a peak around 8 and 9 dpi, both in terms of frequencies as well as absolute numbers (Supplementary Figure S1A and Figure 1A). Within the CD3^+^ T cell compartment, both CD4^+^ and CD8^+^ T cells accumulated at the site of infection in absolute numbers (Figure 1B). However, CD8^+^ T cells accumulated to a greater extent than CD4^+^ T cells in the lungs of IAV-infected mice, reflected by a drop of CD4^+^ T cell frequencies among total CD3^+^ T cells. Importantly, this change in the ratio of CD4^+^ to CD8^+^ T cells in the lungs of IAV-infected mice was more durable, as we did not see a recovery to baseline levels until 21 dpi (Figure 1B).

In dLNs, the frequencies of CD3^+^ T cells constantly, but only mildly, decreased during the course of the IAV infection, while the absolute numbers were only transiently reduced between 7 and 9 dpi (Figure 1C), coinciding with T cell accumulation in infected lungs (Figure 1A) and possibly reflecting T cell recruitment to the site of infection. Only mild changes in the proportion of CD3^+^ T cells were seen during the course of the IAV infection in spleens, which were analyzed as controls (Supplementary Figure S2A). Within the CD3^+^ T cell compartment of dLNs, the ratio of CD4^+^ to CD8^+^ T cells was rather stable and only showed a mild, transient drop in the frequency of CD4^+^ T cells accompanied by a mild, transient increase in the frequency of CD8^+^ T cells at 4 and 5 dpi, both returning to baseline levels already by 6 dpi (Figure 1D). During the entire course of the IAV infection, the absolute numbers of CD4^+^ and CD8^+^ T cells within dLNs showed only mild fluctuations (Figure 1D), and hardly any changes in the ratio of CD4^+^ to CD8^+^ T cells were observed in spleens (Supplementary Figure S2B).

In summary, our data show that the accumulation of CD3^+^ T cells in IAV-infected lungs is accompanied by a durable change in the ratio of CD4^+^ to CD8^+^ T cells, while only mild, transient changes were observed in dLNs.

### 2.2. Two-Step Treg Accumulation Kinetics within IAV-Infected Lungs

**Figure 2:**
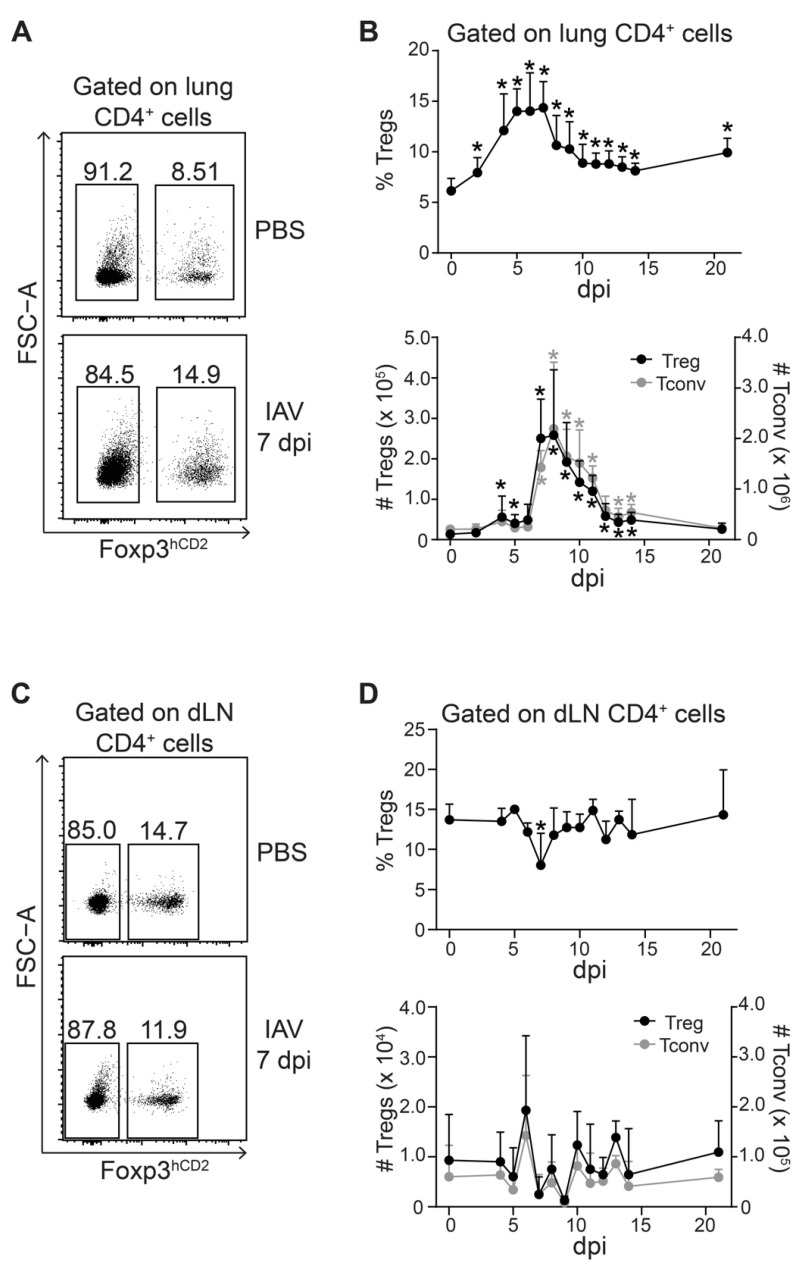
Peak of Treg accumulation coincides with that of Tconv in IAV-infected lungs. Foxp3^hCD2^ × Rag1^GFP^ reporter mice were infected with IAV and analyzed at indicated dpi. Lungs and dLNs were collected and prepared, and single-cell suspensions were restimulated and subsequently analyzed by flow cytometry; (**A**) Representative dot plots depict frequency of Foxp3^hCD2+^ Tregs and Foxp3^hCD2-^ Tconv among CD4^+^ T cells in lungs of PBS-treated and IAV-infected mice (7 dpi); (**B**) Graphs summarize frequencies (top) and absolute numbers (bottom) of Tregs (black filled circles) and Tconv (gray filled circles); (**C**) Representative dot plots depict frequency of Foxp3^hCD2+^ Tregs and Foxp3^hCD2-^ Tconv among CD4^+^ T cells in dLNs of PBS-treated and IAV-infected mice (7 dpi); (**D**) Graphs summarize frequencies (top) and absolute numbers (bottom) of Tregs (black filled circles) and Tconv (gray filled circles). Data were pooled from two to three independent experiments, which included 4–10 mice per group, and presented as mean + SD. Mann–Whitney test was used to calculate statistical significance by comparing values from untreated animals (for lung samples) or PBS-treated animals (for dLN samples) (0 dpi) with values from IAV-infected mice (2 to 21 dpi). Significant changes are indicated by an asterisk.

Tregs were reported to coordinate the early protective immunity to viral infection in a mouse model of Herpes Simplex Virus-2 (HSV-2) infection [26]. Accordingly, they were found to accumulate slightly earlier than conventional T cells (Tconv) in IAV-infected lungs [23]. We revisited this finding by closely following the kinetics of Tregs and Tconv in IAV-infected lungs and dLNs. Within the lungs, the frequencies of Tregs increased continuously and reached a plateau between 5 and 7 dpi (Figure 2A,B). This transient increase was followed by a steady decline until 14 dpi, yet not reaching baseline levels. Interestingly, the accumulation kinetics of absolute Treg numbers showed a two-step pattern. A first small peak was observed at 4 dpi, followed by a short plateau until Day 6 and a subsequent strong increase (>5 fold) at 7 dpi (Figure 2B). Afterwards, the absolute Treg numbers steadily declined until 14 dpi, finally reaching baseline levels at 21 dpi. It is important to note that the absolute numbers of Tconv followed a similar course, showing a highly comparable two-step accumulation kinetic as Tregs (Figure 2A,B).

In dLNs, the frequency of Tregs within the CD4^+^ T cell compartment was rather stable upon infection, and only a mild drop was observed at 7 dpi, which rapidly returned to baseline levels already between 8 and 9 dpi (Figure 2C,D). Similarly, the absolute numbers of Tregs and Tconv within dLNs showed only mild fluctuations during the entire course of the infection not reaching statistical significance (Figure 2D). With the exception of a mild drop in the frequencies of Tregs at 2 dpi, no changes were observed in spleens (Supplementary Figure S2C).

Together, our data suggest a two-step accumulation of Tregs and Tconv within IAV-infected lungs with comparable kinetics. Under the conditions applied in the present study, we did not observe that the accumulation of Tregs within IAV-infected lungs was preceding the accumulation of Tconv.

### 2.3. Synchronized Accumulation of T-Bet Expressing Tregs and Tconv in IAV-Infected Lungs

**Figure 3:**
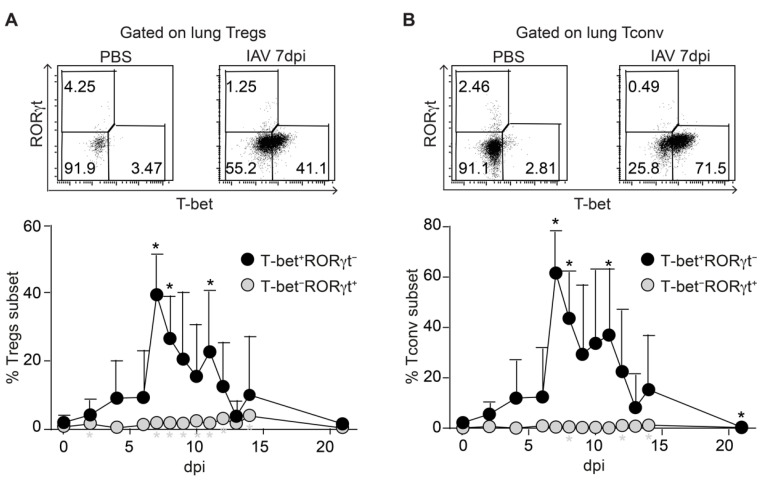
Lung Tregs show a dominant T-bet^+^ phenotype during IAV infection. Foxp3^hCD2^ × Rag1^GFP^ reporter mice were infected with IAV and analyzed at indicated dpi. Lungs were collected and prepared, and single-cell suspensions were restimulated and subsequently analyzed by flow cytometry. (**A**) Representative dot plots depict T-bet and RORγt frequencies among gated Foxp3^hCD2+^ Tregs from lungs of PBS-treated and IAV-infected mice (7 dpi). The graph summarizes frequencies of T-bet^+^RORγt^−^ Tregs (black filled circles) and T-bet^−^RORγt^+^ Tregs (gray filled circles) in lungs collected from IAV-infected mice at the indicated dpi; (**B**) Representative dot plots depict T-bet and RORγt frequencies among gated Tconv from lungs of PBS-treated and IAV-infected mice (7 dpi). The graph summarizes frequencies of T-bet^+^RORγt^−^ Tconv (black filled circles) and T-bet^−^RORγt^+^ Tconv (gray filled circles) in lungs collected from IAV-infected mice at the indicated dpi. Data were pooled from two independent experiments, which included 5–9 mice per group, and presented as mean + SD. Mann–Whitney test was used to calculate statistical significance by comparing values from untreated animals (0 dpi) with values from IAV-infected mice (2 to 21 dpi). Significant changes are indicated by an asterisk.

Next, we analyzed the expression of T-bet and RORγt in T cell subsets isolated from IAV-infected lungs to assess the functional specialization of the Tregs transiently accumulating within IAV-infected lungs. The measurement of RORγt expression was included since a recent report demonstrated the importance of early IL-17A production by tracheal γδ T cells during clearance of IAV infection [27]. The flow cytometric analysis revealed that the frequency of T-bet^+^ Tregs was only slowly increasing during the early stages of the infection (4–6 dpi), followed by a rapid and strong increase at 7 dpi and a subsequent stepwise return to baseline levels until 21 dpi (Figure 3A). In addition, the expression levels of T-bet among T-bet^+^ Tregs, as assessed by the geometric mean fluorescence intensity (gMFI), were also significantly increased in cells recruited at the peak of the response (7 dpi) when compared to earlier stages of the infection (4 dpi) (Supplementary Figure S3A), suggesting a gradual increase in T-bet expression levels in T-bet^+^ cells during the course of the infection. At the same time, only a very small fraction of Tregs within the IAV-infected lungs expressed RORγt at any time point. Remarkably, a similar picture was observed for Tconv, which also showed a strong, but only transient increase in the frequency of T-bet^+^ cells at 7 dpi followed by a stepwise decline, while RORγt^+^ Tconv were essentially absent from IAV-infected lungs (Figure 3B). In line with the findings for T-bet^+^ Tregs, the expression levels of T-bet among T-bet^+^ Tconv were also significantly higher at 7 dpi when compared to 4 dpi (Supplementary Figure S3B). Together, our data indicate that both T-bet^+^ Tregs and T-bet^+^ Tconv accumulate in IAV-infected lungs in a highly synchronized manner, while RORγt was hardly expressed in both CD4^+^ T cell subsets.

To further determine the functional properties of the T cell subsets within the infected lungs, we next analyzed the expression of IL-17A and IFN-γ in Tregs and Tconv by flow cytometry. As expected from the RORγt measurements, the expression of IL-17A was nearly absent in both CD4^+^ T cell populations (Supplementary Figure S4A). However, a significant increase in IFN-γ expression was observed in Tconv at 7 dpi with a marked drop at 14 dpi, whereas the frequencies of IFN-γ^+^ Tregs were rather low with marginal, yet significant alterations at 11 and 14 dpi (Supplementary Figure S4B). In addition, we quantified several cytokines and chemokines within the infected lungs at 7, 9 and 12 dpi. We observed a significant increase in IL-12 (p40), IL-10 and IL-6 at selected time points when compared to PBS-treated controls, while IL-12 (p70) levels remained unchanged (Supplementary Figure S5A). Furthermore, we observed a strong upregulation of the inflammation-driving chemokines CXCL1, CCL2, CCL3, CCL4 and CCL5 at 7 dpi followed by a decline over time (Supplementary Figure S5A). Interestingly, in parallel to our observation of the two-step accumulation of Tregs and Tconv in IAV-infected lungs, we observed high levels of the IFN-γ-induced chemokine IP-10 (CXCL10) in the BALF already at 4 dpi with a further significant increase at 7 dpi (Supplementary Figure S5B). In summary, our data suggest that T-bet expression in Tregs is not triggering a strong IFN-γ expression during IAV infection despite the marked increase in Th1-driving inflammatory mediators within the infected lungs.

### 2.4. Increased Demethylation in Tbx21 and TSDR in T-Bet^+^ Tregs in IAV-Infected Lungs

**Figure 4:**
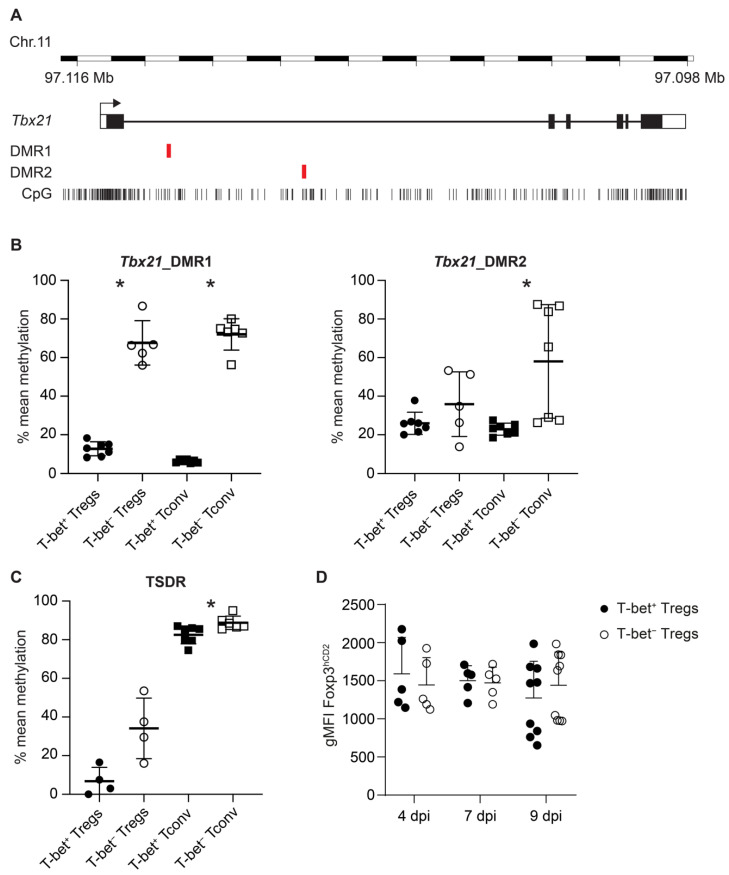
Methylation analysis of *Tbx21* DMRs and the TSDR in T-bet^+^ and T-bet^−^ Tregs and Tconv from IAV-infected lungs. (**A**) Schematic overview of the chromosomal localization, gene structure, position of CpG motifs and analyzed DMR1 and DMR2 of the murine *Tbx21* gene; (**B**,**C**) Lungs from IAV-infected animals were collected and prepared at 10 dpi. T-bet^+^ and T-bet^−^ Treg as well as Tconv subsets were immediately sorted by flow cytometry (see Supplementary Figure S1), and genomic DNA was prepared. The methylation status of *Tbx21*_DMR1, *Tbx21*_DMR2 and the TSDR was determined by pyrosequencing. The graph shows the mean methylation of the indicated regions for individual T-bet^+^ Tregs (black filled circle), T-bet^−^ Tregs (open circle), T-bet^+^ Tconv (black filled square), or T-bet^−^ Tconv (open square) populations. (**D**) Foxp3 expression levels are shown as geometric mean fluorescence intensity (gMFI) of the hCD2 reporter at indicated time points. Each symbol represents data from cells isolated from an individual IAV-infected mouse, and data were pooled from two independent experiments. Mann–Whitney test was used to calculate statistical significance by comparing values from T-bet^+^ Tregs or T-bet^+^ Tconv with T-bet^−^ Tregs or T-bet^−^ Tconv, respectively. Significant changes are indicated by an asterisk.

The lineage-specific transcription factors Foxp3 and T-bet were shown to be regulated by epigenetic mechanisms in Tregs and Th1 cells, respectively, including DNA methylation changes in regulatory elements of their gene loci, which contribute to the stable expression of the transcription factors [11,28,29,30]. To investigate whether T-bet^+^ Tregs accumulating in IAV-infected lungs are epigenetically stabilized, we analyzed the DNA methylation status of two differentially methylated regions (DMRs) within *Tbx21* (Figure 4A) and of the TSDR within *Foxp3* in ex vivo isolated T cell subsets. At 10 dpi, T-bet^+^ and T-bet^−^ Tregs as well as corresponding Tconv subsets were isolated from lungs of IAV-infected mice by flow cytometry. Genomic DNA from the sorted cells was utilized for the DNA methylation analysis by pyrosequencing. The second DMR in *Tbx21* (*Tbx21*_DMR2) was largely demethylated in both T-bet^+^ and T-bet^−^ Tregs, with the latter showing a slightly higher variability (Figure 4B). While T-bet^+^ Tconv also displayed a largely demethylated *Tbx21*_DMR2, in T-bet^−^ Tconv, the DNA methylation status at *Tbx21*_DMR2 was significantly increased, showing, however, a high variability among the analyzed samples. The situation was clearer for the first DMR in *Tbx21* (*Tbx21*_DMR1). Here, we observed an almost complete demethylation in T-bet^+^ Tregs and Tconv, while the corresponding T-bet^−^ subsets were more strongly methylated by a significant margin (Figure 4B). To address the question of whether the epigenetic remodeling of the *Tbx21* locus is already initiated in Tregs and Tconv at earlier time points, namely, during the first and the main peak, we analyzed the methylation status of *Tbx21*_DMR1 at 4 and 7 dpi, respectively. At both time points, the *Tbx21*_DMR1 was already almost fully demethylated in T-bet^+^ Tregs and Tconv, while the corresponding T-bet^−^ subsets were strongly methylated (Supplementary Figure S6). Together, these data suggest that this DMR plays a critical role for the epigenetic control of *Tbx21* expression and that the *Tbx21* locus is already epigenetically remodeled in T-bet^+^ subsets at early time points of the infection.

We next analyzed the methylation status of the TSDR to assess the stability of Foxp3 expression [28], as it had been reported that the expression of T-bet in Tregs can affect the size of the Treg population [31]. Interestingly, the TSDR methylation status was different between T-bet^+^ and T-bet^−^ Tregs. While T-bet^+^ Tregs exhibited an almost completely demethylated TSDR, T-bet^−^ Tregs showed a trend for an increased TSDR methylation (*p* = 0.057) (Figure 4C, Supplementary Table S1), indicating a higher stability of Foxp3 expression in T-bet^+^ Tregs. Yet, the observed epigenetic differences in T-bet^+^ and T-bet^−^ Tregs did not result in differential Foxp3 expression levels 4, 7 or 9 dpi (Figure 4D). As expected, both T-bet^+^ and T-bet^−^ Tconv were strongly methylated at the TSDR, with T-bet^−^ Tconv even showing a significantly stronger methylation (Figure 4C).

Together, our data demonstrated a stronger demethylation of both *Tbx21* (DMR2) and Foxp3 in T-bet^+^ when compared to T-bet^−^ Tregs, indicating a more stable expression of T-bet and Foxp3 under the inflammatory conditions in IAV-infected lungs.

## 3. Discussion

In this study, we characterized the kinetics, phenotype, and epigenetic characteristics of Tregs in the lungs of IAV-infected mice. We observed that the accumulation of CD3^+^ T cells in IAV-infected lungs is accompanied by a durable change in the ratio of CD4^+^ to CD8^+^ T cells, resulting from a strong and long-lasting CD8^+^ T cell response. Furthermore, we demonstrated that the IAV infection induces a robust, but transient Treg response in the infected tissue, which coincides with the peak of Tconv. Interestingly, T-bet but not RORγt is expressed in a substantial proportion of the Tregs within the infected lung. Those T-bet^+^ Tregs displayed a strongly demethylated *Tbx21* locus, and a slightly more pronounced demethylation of the TSDR within the *Foxp3* locus when compared to T-bet^−^ Tregs, suggesting that T-bet^+^ but not T-bet^−^ Tregs are epigenetically stabilized during IAV infection in the lung.

The dynamics of the accumulation of CD4^+^ and CD8^+^ T cells in IAV-infected lungs with a peak at around 8 and 9 dpi observed in the present study are in accordance with previously published reports [25,32]. Interestingly, the ratio of CD4^+^ to CD8^+^ T cells was changed at an early stage of the infection. From 4 dpi onwards, it was dominated by CD8^+^ T cells and did not convert back to baseline levels, highlighting the importance of CD8^+^ T cells for the elimination of IAV-infected cells [33]). A first wave of CD4^+^ and CD8^+^ T cells at 4 dpi was also observed in another study using the IAV strain X31 [34], indicating the pioneer function of T cells for the initiation of further immune cell infiltration, as recently demonstrated in an acute skin inflammation model [35]. Interestingly, in this skin inflammation model, antigen-specific T cells only preferentially accumulated to the inflamed site at early time points, suggesting that the bystander accumulation of non-specific effector/memory T cells at later time points is a general feature in inflammation. Importantly, in the present study, the kinetics of T cells in lung dLNs was different, showing no increase in the frequency of CD3^+^ T cells and a rather stable ratio of CD4^+^ to CD8^+^ T cells, indicating differential accumulation kinetics between the site of infection and the dLNs and emphasizing the relevance of T cells within the infected tissue for the immune defense [36].

Several studies have reported that IAV infection induces a robust, but transient, Treg response within the infected tissue [23,24,37]. The finding that the Treg peak was preceding that of effector T cells [23] led to the conclusion that Tregs might facilitate the entry of effector cells into the site of infection to coordinate the early protective immunity against pathogens as previously demonstrated for HSV-2 infection [26]. However, in the present study, we did not observe an earlier accumulation of Tregs in IAV-infected lungs, but rather noticed a highly synchronized, two-step accumulation of Tregs and Tconv. This apparently contradictory finding does not exclude that the first Tregs entering the IAV-infected lungs are facilitating the entry of effector cells into the infected site to coordinate the early protective immunity against IAV. Yet, it is also possible that those Tregs, which are migrating into the IAV-infected lungs at an early stage of the infection, mainly contribute to the prevention of over-shooting immune responses and excessive immunopathology as reported before [37]. The precise functional role of the first Tregs entering the IAV-infected lungs needs to be unraveled in future studies, and the impact the IAV-induced thymus atrophy and the concomitantly altered thymic Treg generation [38] have in this process also has to be determined.

Numerous studies have highlighted that Tregs residing in non-lymphoid tissues not only exert immunosuppressive but also various non-canonical functions, including the promotion of tissue repair mediated by amphiregulin [39]. During IAV infection, the selective Treg deficiency of amphiregulin resulted in severe acute lung damage without any detectable alterations in Treg suppressor function, antiviral immune response, and viral load, demonstrating the direct and non-redundant role of Tregs in tissue repair during infectious lung injury [40]. Tissue-resident Tregs are known to acquire their unique phenotypic and functional properties in a stepwise process by transitioning through a precursor stage in secondary lymphoid organs and terminal differentiation in their respective tissues [41,42]. While our data indicate that T-bet can swiftly be co-expressed by Tregs accumulating in lungs during IAV infection, they do not allow for dissecting if T-bet is induced on tissue-resident and/or newly recruited Tregs.

Under steady-state conditions, T-bet expression in Tregs seems to be dispensable [13], in contrast to other transcription factors such as IRF4 or STAT3, whose deficiencies in Tregs result in spontaneous immune-mediated inflammation [43,44]. However, under Th1-favoring conditions, when T-bet is rapidly induced in Tregs, the impaired capacity of T-bet-deficient Tregs to co-localize with Th1 cells and specifically suppress their responses is readily uncovered [13,17,18]. Thus, it is highly likely that during IAV-induced infection, and also during other Th1-dominated infections such as lymphocytic choriomeningitis virus (LCMV), vaccinia virus (VV), and *Legionella pneumophila*, which all show a similar transient accumulation of T-bet^+^ Tregs within the infected tissue [18], T-bet^+^ Tregs control the Th1-driven immune response. However, whether T-bet^+^ Tregs in IAV-infected lungs also promote the generation of CD8^+^ T_RM_ cells by providing TGF-β as recently reported [19] or contribute to tissue repair following the inflammatory response remains to be determined.

The molecular factors mediating the induction of T-bet in Tregs during Th1 responses are incompletely understood. Previously, it was reported that T-bet expression in Tregs is induced by an IFN-γ- and Stat1-dependent, but IL 12-independent, signaling pathway [13]. In addition, IL-27 was shown to promote the expression of T-bet in Tregs, particularly at mucosal sites; however, transcriptional profiling studies revealed that T-bet^+^ Tregs generated upon exposure to either IFN-γ or IL-27 are different, suggesting that these two cytokines have different roles in Treg biology [45]. It is tempting to speculate that T-bet^−^ Tregs and Tconv would readily upregulate T-bet expression within the lung if stimulated appropriately as findings of the present study have demonstrated a partially demethylated *Tbx21* locus for both lung-derived T cell subsets, suggesting a ‘poised’ state that is different from that of naïve CD4^+^ T cells which display an almost completely methylated *Tbx21* locus [30]. In addition to cytokine-driven signals, signals via the TCR are also critically required for the epigenetic imprinting of T cells, as recently demonstrated for the development of Foxp3^+^ Tregs [46]. Moreover, an involvement of TCR signaling strength was underpinned by the finding that the expression level of T-bet in in vitro stimulated Tregs was directly dependent on the TCR trigger, and that the TCR repertoire between CXCR3^+^ and CXCR3^−^ Tregs differs [47]. Therefore, it is likely that combined signaling events regulate the epigenetic modification of the *Tbx21* locus in Tregs in an inflammatory environment, and it is also plausible that specific TCR signals received by T-bet^+^ Tregs contribute to the slightly more pronounced demethylation of the TSDR. Yet, we did not observe higher Foxp3 expression levels among T-bet^+^ Tregs. Further research is needed to unravel the exact molecular mechanisms and the role of the cytokines IFN-γ and IL-27 in combination with TCR signaling to mediate the specific demethylation of the *Tbx21* locus in Tregs.

The inflammatory environment induced by the IAV infection in mice is probably counteracting to the peripheral de novo induction of Tregs [48] and arguing against a parallel induction of regulatory- and Th1-driven transcription to generate T-bet^+^ Tregs. Another origin of the T-bet^+^ Tregs could be Th1 cells, although they were described as a rather stable Th lineage with limited plasticity [49]. Yet, a recent publication described the induction of Foxp3 expression in resting IFN-γ^+^ Th1 cells in vitro [50]. However, a link to the observation in IAV-infected animals seem to be improper, as in the in vitro culture system, converted Th1 cells originate from a cytokine-free resting phase, show no signs of epigenetic regulation in the *Foxp3* gene locus and continuously express IFN-γ. Therefore, it is most likely that in the present study T-bet^+^ Tregs originate from T-bet^−^ Tregs and partially adopt Th1-driven molecules such as CXCR3 but not IFN-γ.

T-bet^+^ Tregs isolated from IAV-infected lungs showed an almost completely demethylated TSDR, demonstrating a high stability of the Treg phenotype within this inflammatory environment [8,51]. Furthermore, the *Tbx21* locus was also strongly demethylated, already at early time points, indicating a stable T-bet expression among the first wave of immigrating/appearing T-bet^+^ Tregs. Indeed, such an intrinsic stability of T-bet^+^ Tregs was recently reported in a Th1-driven bacterial infection model [17]. However, it is still controversially discussed whether functional Treg specialization is paralleled by memory generation and whether Tregs can long-lastingly acquire phenotypic or functional changes upon inflammation or antigen-specific activation. One study had reported that although inflammation-experienced Tregs can be maintained long term, they reversed many activation-induced transcriptional and epigenomic changes and lost their enhanced suppressive function over time [52]. The contrary was observed for Tregs induced upon IAV infection [37]. Here, adoptive transfer of IAV-induced memory but not naïve Tregs significantly attenuated body weight loss, lung pathology and immune cell infiltration into IAV-infected lungs, suggesting that Tregs can differentiate into cells with enhanced suppressive function upon infection [37]. Yet, this concept could not be generalized as adoptive transfer of infection-experienced Tregs generated upon acute LCMV or VV infections did not result in an enhanced suppressive capacity upon re-challenge when compared to the adoptive transfer of naïve Tregs [53], suggesting that Tregs do not always form functional memory upon viral infections. Future studies need to unravel if systemic immune responses differ from local ones in their capacity to generate functional memory among Tregs, and if the known impact of tissues on the unique epigenetic signatures of tissue-resident Tregs [42] is involved in the stabilization of functional properties in Tregs to prevent overt immunopathology in recurrent infections.

## 4. Materials and Methods

### 4.1. Mice and Virus

Foxp3^hCD2^ × Rag1^GFP^ reporter mice [51,54] on a C57BL/6 background or wild-type C57BL/6 mice were bred and maintained at the animal facility of the Helmholtz Centre for Infection Research (Braunschweig, Germany). Importantly, in the reporter mice, hCD2 expression accurately correlates with Foxp3 expression, even upon in vitro stimulation [51]. All mice were housed under specific pathogen-free conditions in isolated, ventilated cages, and handled in accordance with good animal practice as defined by FELASA (Federation of European Laboratory Animal Science Associations) and the national animal welfare body GV-SOLAS (Society for Laboratory Animal Science). All animal experiments were approved by the Lower Saxony Committee on the Ethics of Animal Experiments as well as the responsible state office (Lower Saxony State Office of Consumer Protection and Food Safety) under the permit number 33.19-42502-04-15/2058 (date of approval: 30 January 2016) or 33.19-42502-04-16/2319 (date of approval: 4 November 2016). In all experiments, female mice at the age of six weeks were used, and for the infection experiments, the mouse-adapted strain PR8M of IAV H1N1 was used. The virus was grown on the MDCK cell line, harvested as a supernatant of the cultures, aliquoted and stored at −80 °C. A total of 666 focus-forming units (ffu)/20 µL phosphate-buffered saline (PBS) (Gibco/Fisher Scientific, Schwerte, Germany) were given intranasally to anesthetized mice.

### 4.2. Isolation of Cells from dLNs, Spleens and Lungs

Single-cell suspensions were prepared from dLNs or spleens by using a 100 µm cell strainer, and cleared from erythrocytes by incubation with ammonium chloride buffer at room temperature for 3 min. Isolation of cells from the lungs of IAV-infected or uninfected control mice was performed according to a protocol modified from Wilk and Schughart [55]. Briefly, mice were perfused with PBS until lungs became pale pink. Then, lungs were collected and cut into small pieces, which were digested using 0.2 mg/mL collagenase D (Roche, Basel, Switzerland) and 10 μg/mL DNaseI (Roche). To purify lymphocytes, lysates were mixed with 40% Percoll (Fisher Scientific, Schwerte, Germany) and layered on top of a 70% Percoll layer. The mixture was centrifuged at 780× *g* at 4 °C for 30 min without brake. Finally, lymphocytes were collected from the interface between the two layers.

### 4.3. Antibodies and Flow Cytometry

Flow cytometric analysis was performed as described recently [56]. In brief, single-cell suspensions were prepared from dLNs, spleens and lungs. If not stated otherwise, cells were restimulated by incubation with phorbol 12-myristate 13-acetate (PMA; 10 ng/mL, 4 h; Sigma-Aldrich, St. Louis, MO, USA) and ionomycin (0.5 µg/mL, 4 h; Sigma-Aldrich) with the addition of Brefeldin A (10 µg/mL; Sigma Aldrich) for the final two hours. Subsequently, cells were labeled directly with the following fluorochrome-conjugated anti-mouse antibodies purchased from either BioLegend (San Diego, CA, USA), BD Biosciences (Franklin Lakes, NJ, USA) or eBioscience/Thermo Fisher Scientific (San Diego, CA, USA): CD3-PE-Cy7 (17A2), CD4-BV605 (RM4-5), CD8α-V500 (53-6.7), human CD2-APC (RPA-2.10), human CD2-PerCP-Cy5.5 (RPA-2.10), human CD2-BV421 (RPA-2.10), RORγt-BV421 (Q31-378), T-bet-PE (eBio4B10 (4B10)), IL-17A-APC (TC11-18H10.1), and IFN-γ-FITC (XMG1.2). Intracellular staining of cytokines and transcription factors was performed by employing the eBioscience™ Intracellular Fixation and Permeabilization kit (Thermo Fisher Scientific). Dead cells were excluded by using the LIVE/DEAD™ Fixable Blue stain kit (Invitrogen/Thermo Fisher Scientific, Waltham, MA, USA). For analysis of transcription factor expression in lung Tregs, the cells were gated according to the expression level of T-bet and RORγt among the CD3^+^CD4^−^CD8^−^ population, which showed a clearly demarcated T-bet^+^ and RORγt^+^ population. Flow cytometric analysis was performed on LSRFortessa™ flow cytometer (BD Biosciences), and data were analyzed with FlowJo software (BD Biosciences).

### 4.4. Cell Sorting

Single-cell suspensions from digested lungs taken from IAV-infected mice were immediately stained with the following fluorochrome-conjugated antibodies (purchased from either BioLegend, BD Biosciences or eBioscience/Thermo Fisher Scientific): CD3-PE-Cy7 (17A2), CD4-BV605 (RM4-5), CD8α-V500 (53-6.7), human CD2-PerCP-Cy5.5 (RPA-2.10). Intracellular staining was performed as mentioned above, and Tregs and Tconv were sorted as either CD3^+^CD4^+^CD8^−^Foxp3^hCD2+^T-bet^+^, CD3^+^CD4^+^CD8^−^Foxp3^hCD2+^T-bet^−^, CD3^+^CD4^+^CD8^−^Foxp3^hCD2−^T-bet^+^, or CD3^+^CD4^+^CD8^−^Foxp3^hCD2−^T-bet^−^ (Supplementary Figure S1). BD FACSAria™ Fusion (BD Biosciences) was employed for cell sorting.

### 4.5. DNA Methylation Analysis

Genomic DNA was isolated from sorted cells using the NucleoSpin Tissue kit (Macherey-Nagel, Dueren, Germany) in accordance with the manufacturer’s protocol with minor modifications. In an additional step, Chelex-100 beads (Biorad, Hercules, CA, USA) were added after the lysis step and incubated at 95 °C for 15 min in a shaker to release the formaldehyde-induced crosslinks. Beads were spun down, the supernatant was transferred to a new tube and complemented with an adjusted amount of 99.8% ethanol (Merck, Darmstadt, Germany). The column-based purification was completed according to the manufacturer’s protocol. The DNA concentration was assessed with a Nanodrop 1000 spectrophotometer (Peqlab, Erlangen, Germany). Methylation analysis of two differentially methylated regions in *Tbx21* (*Tbx21*_DMR1, *Tbx21*_DMR2) and the Treg-specific demethylated region (TSDR) within the *Foxp3* gene locus was performed by pyrosequencing: Bisulfite conversion was achieved using the EZ DNA Methylation Kit (Zymo Research, Irvine, CA, USA) according to the standard procedure. *Tbx21*_DMR1, *Tbx21*_DMR2 or the TSDR were amplified by PCR using bisulfite-converted DNA, the primers mTbx21-1-for (5′-TATTTAGAGAAGGATTGGGGGAGT-3′) and mTbx21-1-rev (5′-bio-CCCAAAAAACACTACTAAAATATATTCC-3′) or mTbx21-2-for (5′-bio-AATAGGAAGAAGAGTTTGGAGTTTT-3′) and mTbx21-2-rev (5′-ACCAAAAAAATAAAAATTACCAACAATTAA-3′) or mTSDR-for (5′-bio-TAAGGGGGTTTTAATATTTATGAGGTTT-3′) and mTSDR-rev (5′-CTAAACTAACCAACCAACTTCCTA-3′) and the ZymoTaq PreMix (Zymo Research) following the manufacturer’s protocol. The amplificates were sequenced by pyrosequencing, using the sequencing primer mTbx21-1-seq1 (5′-GGATTGGGGGAGTAGA-3′), mTbx21-2-seq1 (5′-CTCTTACCTACTAAAAAAACCTTT-3′), mTbx21-2-seq2 (5′-ACTACACACTAACTAACTC-3′), mTSDR-seq1 (5′-ACCCAAATAAAATAATATAAATACT-3′) mTSDR-seq2 (5′-ATCTACCCCACAAATTT-3′), or mTSDR-seq3 (5′-AACCAAATTTTTCTACCATT-3′) on a Pyromark Q24 (Qiagen, Hilden, Germany) and analyzed following the manufacturer’s instructions. Characterized CpG motifs are located on chromosome 11:97109288-97109359, 97113285-97113341 (*Tbx21*) or chromosome X:7583986-7584149 (*Foxp3*) (GRCm38.p6).

### 4.6. Cytokine/Chemokine Measurement

Lungs from PBS-treated and IAV-infected mice were prepared, homogenized and lysed using the Bio-Plex Cell Lysis Kit according to the manufacturer’s instructions (Bio-Rad Laboratories). Cytokine/chemokine measurement with Bio-Plex Pro Mouse Cytokine 23-plex Assay Kit (Bio-Rad Laboratories) was performed as described recently [38].

For isolation of the bronchoalveolar lavage fluid (BALF), the trachea of IAV-infected mice were punctured and a vein catheter was inserted. BALF was obtained by flushing the lungs using 1 mL PBS and cleared by centrifugation (420× *g*, 10 min). Levels of IP-10 (CXCL10) were determined by using the Mouse CXCL10/IP-10/CRG-2 DuoSet ELISA (R&D Systems, Minneapolis, MN, USA) according to the manufacturer’s instructions.

### 4.7. Statistical Analysis

The GraphPad Prism v7.0 (GraphPad Software, San Diego, CA, USA) was used to perform all statistical analyses. Data are presented as mean + standard deviation (SD) or mean ± SD. For the comparison of unmatched groups, the two-tailed Mann–Whitney test was performed and the *p*-values were calculated with the long-rank test (Mantel–Cox). For the statistical analysis of the CXCL10 concentrations obtained from BALF, a two-tailed, unpaired t test with Welch’s correction was performed. A *p*-value below 0.05 was considered significant and is indicated in the figures by a single asterisk to increase readability. The detailed classification of differences in cell frequencies, numbers, methylation values into * *p* < 0.05, ** *p* < 0.01, *** *p* < 0.001, **** *p* < 0.0001, and ns (not significant) can be found in Supplementary Table S1.

## Data Availability

Not applicable.

## Supplementary Materials

The following are available online at https://www.mdpi.com/article/10.3390/ijms22147522/s1.

## References

[1] Brunkow M.E., Jeffery E.W., Hjerrild K.A., Paeper B., Clark L.B., Yasayko S.A., Wilkinson J.E., Galas D., Ziegler S.F., Ramsdell F. (2001). Disruption of a new forkhead/winged-helix protein, scurfin, results in the fatal lymphoproliferative disorder of the scurfy mouse. Nat. Genet..

[2] Bennett C.L., Christie J., Ramsdell F., Brunkow M.E., Ferguson P.J., Whitesell L., Kelly T.E., Saulsbury F.T., Chance P.F., Ochs H.D. (2001). The immune dysregulation, polyendocrinopathy, enteropathy, X-linked syndrome (IPEX) is caused by mutations of FOXP3. Nat. Genet..

[3] Kim J.M., Rasmussen J.P., Rudensky A.Y. (2007). Regulatory T cells prevent catastrophic autoimmunity throughout the lifespan of mice. Nat. Immunol..

[4] Lahl K., Loddenkemper C., Drouin C., Freyer J., Arnason J., Eberl G., Hamann A., Wagner H., Huehn J., Sparwasser T. (2007). Selective depletion of Foxp3^+^ regulatory T cells induces a scurfy-like disease. J. Exp. Med..

[5] Hori S., Sakaguchi S. (2004). Foxp3: A critical regulator of the development and function of regulatory T cells. Microbes Infect..

[6] Klein L., Robey E.A., Hsieh C.S. (2019). Central CD4^+^ T cell tolerance: Deletion versus regulatory T cell differentiation. Nat. Rev. Immunol..

[7] Pasztoi M., Pezoldt J., Huehn J. (2015). Microenvironment Matters: Unique Conditions Within Gut-Draining Lymph Nodes Favor Efficient De Novo Induction of Regulatory T Cells. Prog. Mol. Biol. Transl. Sci..

[8] Floess S., Freyer J., Siewert C., Baron U., Olek S., Polansky J., Schlawe K., Chang H.D., Bopp T., Schmitt E. (2007). Epigenetic control of the foxp3 locus in regulatory T cells. PLoS Biol..

[9] Toker A., Engelbert D., Garg G., Polansky J.K., Floess S., Miyao T., Baron U., Duber S., Geffers R., Giehr P. (2013). Active demethylation of the Foxp3 locus leads to the generation of stable regulatory T cells within the thymus. J. Immunol..

[10] Delacher M., Imbusch C.D., Weichenhan D., Breiling A., Hotz-Wagenblatt A., Trager U., Hofer A.C., Kagebein D., Wang Q., Frauhammer F. (2017). Genome-wide DNA-methylation landscape defines specialization of regulatory T cells in tissues. Nat. Immunol..

[11] Schmidl C., Delacher M., Huehn J., Feuerer M. (2018). Epigenetic mechanisms regulating T-cell responses. J. Allergy Clin. Immunol..

[12] Lochner M., Peduto L., Cherrier M., Sawa S., Langa F., Varona R., Riethmacher D., Si-Tahar M., Di Santo J.P., Eberl G. (2008). In vivo equilibrium of proinflammatory IL-17^+^ and regulatory IL-10^+^ Foxp3^+^ RORgamma t^+^ T cells. J. Exp. Med..

[13] Koch M.A., Tucker-Heard G., Perdue N.R., Killebrew J.R., Urdahl K.B., Campbell D.J. (2009). The transcription factor T-bet controls regulatory T cell homeostasis and function during type 1 inflammation. Nat. Immunol..

[14] Wang Y., Su M.A., Wan Y.Y. (2011). An essential role of the transcription factor GATA-3 for the function of regulatory T cells. Immunity.

[15] Wohlfert E.A., Grainger J.R., Bouladoux N., Konkel J.E., Oldenhove G., Ribeiro C.H., Hall J.A., Yagi R., Naik S., Bhairavabhotla R. (2011). GATA3 controls Foxp3^+^ regulatory T cell fate during inflammation in mice. J. Clin. Investig..

[16] Chaudhry A., Rudensky A.Y. (2013). Control of inflammation by integration of environmental cues by regulatory T cells. J. Clin. Investig..

[17] Levine A.G., Mendoza A., Hemmers S., Moltedo B., Niec R.E., Schizas M., Hoyos B.E., Putintseva E.V., Chaudhry A., Dikiy S. (2017). Stability and function of regulatory T cells expressing the transcription factor T-bet. Nature.

[18] Littringer K., Moresi C., Rakebrandt N., Zhou X., Schorer M., Dolowschiak T., Kirchner F., Rost F., Keller C.W., McHugh D. (2018). Common Features of Regulatory T Cell Specialization During Th1 Responses. Front. Immunol..

[19] Ferreira C., Barros L., Baptista M., Blankenhaus B., Barros A., Figueiredo-Campos P., Konjar S., Laine A., Kamenjarin N., Stojanovic A. (2020). Type 1 Treg cells promote the generation of CD8^+^ tissue-resident memory T cells. Nat. Immunol..

[20] Leon B., Bradley J.E., Lund F.E., Randall T.D., Ballesteros-Tato A. (2014). FoxP3^+^ regulatory T cells promote influenza-specific Tfh responses by controlling IL-2 availability. Nat. Commun..

[21] Hornick E.E., Zacharias Z.R., Legge K.L. (2019). Kinetics and Phenotype of the CD4 T Cell Response to Influenza Virus Infections. Front. Immunol..

[22] Miyauchi K. (2017). Helper T Cell Responses to Respiratory Viruses in the Lung: Development, Virus Suppression, and Pathogenesis. Viral Immunol..

[23] Betts R.J., Prabhu N., Ho A.W., Lew F.C., Hutchinson P.E., Rotzschke O., Macary P.A., Kemeny D.M. (2012). Influenza A virus infection results in a robust, antigen-responsive, and widely disseminated Foxp3^+^ regulatory T cell response. J. Virol..

[24] Bedoya F., Cheng G.S., Leibow A., Zakhary N., Weissler K., Garcia V., Aitken M., Kropf E., Garlick D.S., Wherry E.J. (2013). Viral antigen induces differentiation of Foxp3^+^ natural regulatory T cells in influenza virus-infected mice. J. Immunol..

[25] Roman E., Miller E., Harmsen A., Wiley J., Von Andrian U.H., Huston G., Swain S.L. (2002). CD4 effector T cell subsets in the response to influenza: Heterogeneity, migration, and function. J. Exp. Med..

[26] Lund J.M., Hsing L., Pham T.T., Rudensky A.Y. (2008). Coordination of early protective immunity to viral infection by regulatory T cells. Science.

[27] Palomino-Segura M., Latino I., Farsakoglu Y., Gonzalez S.F. (2020). Early production of IL-17A by gammadelta T cells in the trachea promotes viral clearance during influenza infection in mice. Eur. J. Immunol..

[28] Huehn J., Polansky J.K., Hamann A. (2009). Epigenetic control of FOXP3 expression: The key to a stable regulatory T-cell lineage?. Nat. Rev. Immunol..

[29] Tripathi S.K., Lahesmaa R. (2014). Transcriptional and epigenetic regulation of T-helper lineage specification. Immunol. Rev..

[30] Mazzoni A., Santarlasci V., Maggi L., Capone M., Rossi M.C., Querci V., De Palma R., Chang H.D., Thiel A., Cimaz R. (2015). Demethylation of the RORC2 and IL17A in human CD4^+^ T lymphocytes defines Th17 origin of nonclassic Th1 cells. J. Immunol..

[31] Tahara M., Kondo Y., Yokosawa M., Tsuboi H., Takahashi S., Shibayama S., Matsumoto I., Sumida T. (2015). T-bet regulates differentiation of forkhead box protein 3^+^ regulatory T cells in programmed cell death-1-deficient mice. Clin. Exp. Immunol..

[32] Sun J., Madan R., Karp C.L., Braciale T.J. (2009). Effector T cells control lung inflammation during acute influenza virus infection by producing IL-10. Nat. Med..

[33] Guo X.J., Thomas P.G. (2017). New fronts emerge in the influenza cytokine storm. Semin. Immunopathol..

[34] Richter M., Ray S.J., Chapman T.J., Austin S.J., Rebhahn J., Mosmann T.R., Gardner H., Kotelianski V., deFougerolles A.R., Topham D.J. (2007). Collagen distribution and expression of collagen-binding alpha1beta1 (VLA-1) and alpha2beta1 (VLA-2) integrins on CD4 and CD8 T cells during influenza infection. J. Immunol..

[35] Ghani S., Feuerer M., Doebis C., Lauer U., Loddenkemper C., Huehn J., Hamann A., Syrbe U. (2009). T cells as pioneers: Antigen-specific T cells condition inflamed sites for high-rate antigen-non-specific effector cell recruitment. Immunology.

[36] Mueller S.N., Mackay L.K. (2016). Tissue-resident memory T cells: Local specialists in immune defence. Nat. Rev. Immunol..

[37] Lu C., Zanker D., Lock P., Jiang X., Deng J., Duan M., Liu C., Faou P., Hickey M.J., Chen W. (2019). Memory regulatory T cells home to the lung and control influenza A virus infection. Immunol. Cell Biol..

[38] Elfaki Y., Robert P.A., Binz C., Falk C.S., Bruder D., Prinz I., Floess S., Meyer-Hermann M., Huehn J. (2021). Influenza A virus-induced thymus atrophy differentially affects dynamics of conventional and regulatory T cell development in mice. Eur. J. Immunol..

[39] Panduro M., Benoist C., Mathis D. (2016). Tissue Tregs. Annu. Rev. Immunol..

[40] Arpaia N., Green J.A., Moltedo B., Arvey A., Hemmers S., Yuan S., Treuting P.M., Rudensky A.Y. (2015). A Distinct Function of Regulatory T Cells in Tissue Protection. Cell.

[41] Li C., DiSpirito J.R., Zemmour D., Spallanzani R.G., Kuswanto W., Benoist C., Mathis D. (2018). TCR Transgenic Mice Reveal Stepwise, Multi-site Acquisition of the Distinctive Fat-Treg Phenotype. Cell.

[42] Delacher M., Imbusch C.D., Hotz-Wagenblatt A., Mallm J.P., Bauer K., Simon M., Riegel D., Rendeiro A.F., Bittner S., Sanderink L. (2020). Precursors for Nonlymphoid-Tissue Treg Cells Reside in Secondary Lymphoid Organs and Are Programmed by the Transcription Factor BATF. Immunity.

[43] Zheng Y., Chaudhry A., Kas A., deRoos P., Kim J.M., Chu T.T., Corcoran L., Treuting P., Klein U., Rudensky A.Y. (2009). Regulatory T-cell suppressor program co-opts transcription factor IRF4 to control T(H)2 responses. Nature.

[44] Chaudhry A., Rudra D., Treuting P., Samstein R.M., Liang Y., Kas A., Rudensky A.Y. (2009). CD4^+^ regulatory T cells control TH17 responses in a Stat3-dependent manner. Science.

[45] Hall A.O., Beiting D.P., Tato C., John B., Oldenhove G., Lombana C.G., Pritchard G.H., Silver J.S., Bouladoux N., Stumhofer J.S. (2012). The cytokines interleukin 27 and interferon-gamma promote distinct Treg cell populations required to limit infection-induced pathology. Immunity.

[46] Ohkura N., Hamaguchi M., Morikawa H., Sugimura K., Tanaka A., Ito Y., Osaki M., Tanaka Y., Yamashita R., Nakano N. (2012). T cell receptor stimulation-induced epigenetic changes and Foxp3 expression are independent and complementary events required for Treg cell development. Immunity.

[47] Sprouse M.L., Scavuzzo M.A., Blum S., Shevchenko I., Lee T., Makedonas G., Borowiak M., Bettini M.L., Bettini M. (2018). High self-reactivity drives T-bet and potentiates Treg function in tissue-specific autoimmunity. JCI Insight.

[48] Wei J., Duramad O., Perng O.A., Reiner S.L., Liu Y.J., Qin F.X. (2007). Antagonistic nature of T helper 1/2 developmental programs in opposing peripheral induction of Foxp3^+^ regulatory T cells. Proc. Natl. Acad. Sci. USA.

[49] Crotty S. (2018). Do Memory CD4 T Cells Keep Their Cell-Type Programming: Plasticity versus Fate Commitment? Complexities of Interpretation due to the Heterogeneity of Memory CD4 T Cells, Including T Follicular Helper Cells. Cold Spring Harb. Perspect. Biol..

[50] Kanamori M., Nakatsukasa H., Ito M., Chikuma S., Yoshimura A. (2018). Reprogramming of Th1 cells into regulatory T cells through rewiring of the metabolic status. Int. Immunol..

[51] Miyao T., Floess S., Setoguchi R., Luche H., Fehling H.J., Waldmann H., Huehn J., Hori S. (2012). Plasticity of Foxp3^+^ T cells reflects promiscuous Foxp3 expression in conventional T cells but not reprogramming of regulatory T cells. Immunity.

[52] van der Veeken J., Gonzalez A.J., Cho H., Arvey A., Hemmers S., Leslie C.S., Rudensky A.Y. (2016). Memory of Inflammation in Regulatory T Cells. Cell.

[53] Rost F., Lambert K., Rakebrandt N., Joller N. (2020). Preceding Viral Infections Do Not Imprint Long-Term Changes in Regulatory T Cell Function. Sci. Rep..

[54] Kuwata N., Igarashi H., Ohmura T., Aizawa S., Sakaguchi N. (1999). Cutting edge: Absence of expression of RAG1 in peritoneal B-1 cells detected by knocking into RAG1 locus with green fluorescent protein gene. J. Immunol..

[55] Wilk E., Schughart K. (2012). The Mouse as Model System to Study Host-Pathogen Interactions in Influenza A Infections. Curr. Protoc. Mouse Biol..

[56] Cossarizza A., Chang H.D., Radbruch A., Acs A., Adam D., Adam-Klages S., Agace W.W., Aghaeepour N., Akdis M., Allez M. (2019). Guidelines for the use of flow cytometry and cell sorting in immunological studies (second edition). Eur. J. Immunol..

