# Supplementary material for: Tbx21 and Foxp3 Are Epigenetically Stabilized in T-Bet+ Tregs That Transiently Accumulate in Influenza A Virus-Infected Lungs
Source: Int J Mol Sci. 2021 Jul 14;22(14):7522. doi: 10.3390/ijms22147522 (PMC8307036; doi:10.3390/ijms22147522)
Supplement: Supplementary file 1 [file ijms-22-07522-s001.zip › ijms-1298654-SI.pdf]

Supplementary Materials

Tbx21 and *Foxp3* are epigenetically stabilized in T-bet^{+} Tregs that transiently accumulate in influenza A virus-infected lungs

Yassin Elfaki, Juhao Yang, Julia Boehme, Kristin Schultz, Dunja Bruder, Christine S. Falk, Jochen Huehn, and Stefan Floess

Supplementary Figure S1

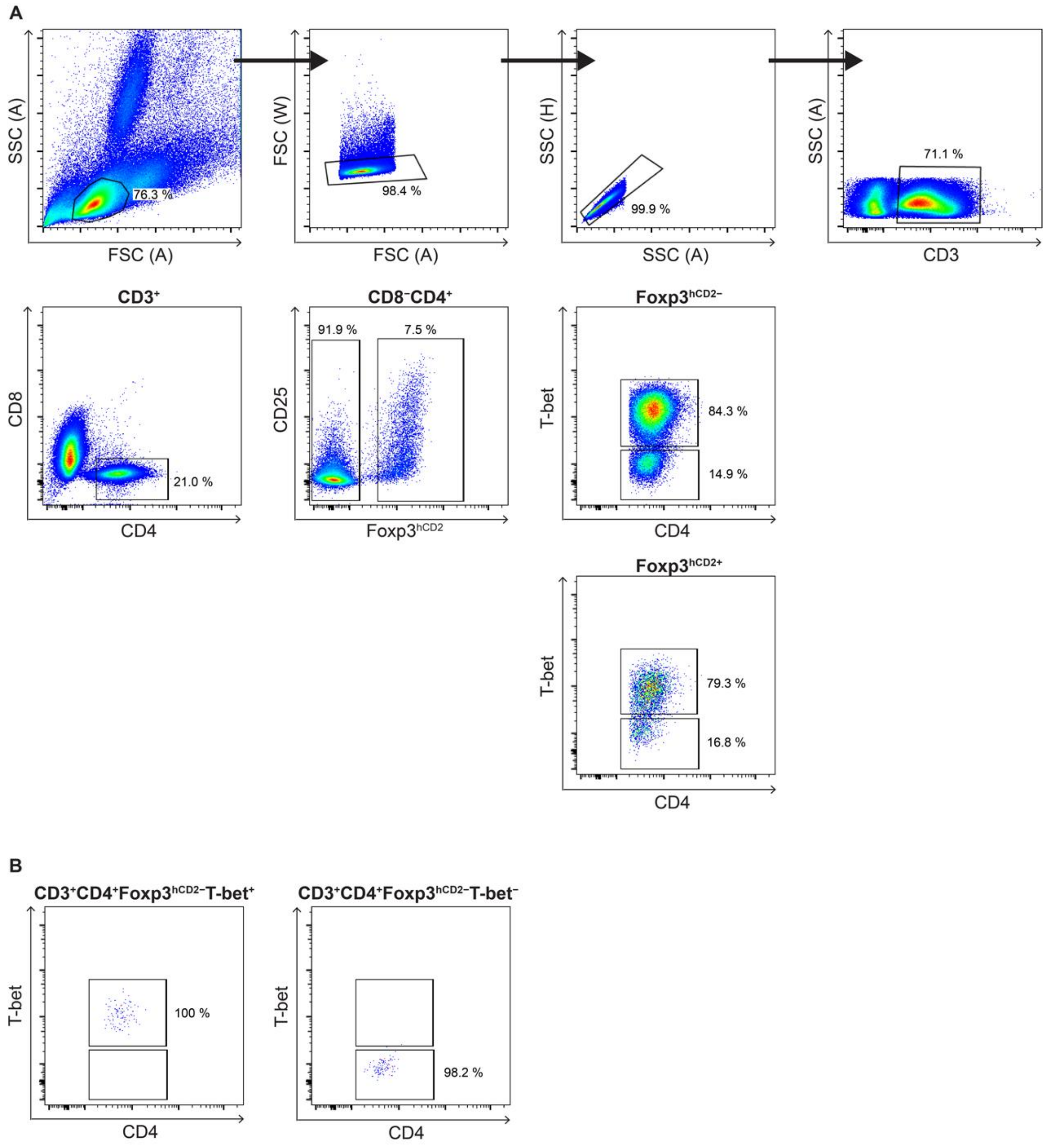

Figure S1: Sorting of T-bet$^-$ and T-bet$^+$ Tregs and Tconv from IAV-infected lungs. Foxp3^{hCD2} x Rag1^{GFP} reporter mice were infected with IAV, and lungs were collected and prepared at 10 dpi. Single-cell suspensions were immediatedly stained and sorted as either CD3$^+$CD4$^+$CD8$^-$Foxp3^{hCD2+}T-bet$^+$, CD3$^+$CD4$^+$CD8$^-$Foxp3^{hCD2+}T-bet$^-$, CD3$^+$CD4$^+$CD8$^-$Foxp3^{hCD2-}T-bet$^+$, or CD3$^+$CD4$^+$CD8$^-$Foxp3^{hCD2-}T-bet$^-$. (**a**) Pseudocolor plots depict the markers used for sorting. The black-rimmed areas indicate the sort gates, the numbers indicate the corresponding frequencies. One representative sample is shown (n=7). (**b**) Representative dot plots depict purity of sorted T-bet$^+$ (left) and T-bet$^-$ (right) Tconv (mean purity of T-bet$^+$ cells: 99.57 %; mean purity of T-bet$^-$ cells: 97.96 %).

Supplementary Figure S2

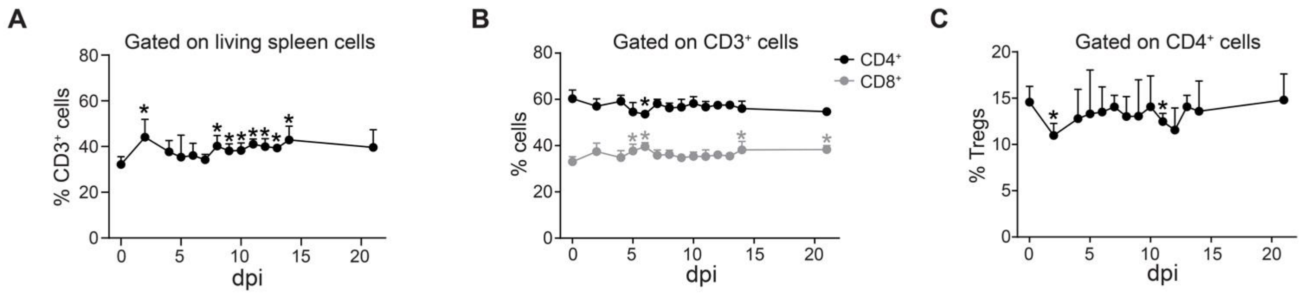

Figure S2: Kinetics of T cell accumulation in spleen during IAV infection. Foxp3^{hCD2} x Rag1^{GFP} reporter mice were infected with IAV and analyzed at indicated dpi. Spleens were collected and prepared, and single-cell suspensions were restimulated and subsequently analyzed by flow cytometry. (**a**) Graph summarizes frequency of CD3^{+} cells, gated on living splenocytes. (**b**) Frequencies of CD4^{+} (black filled circles) and CD8^{+} T cells (grey filled circles) gated on CD3^{+} splenocytes, and (**c**) frequency of Foxp3^{+} Tregs gated on CD4^{+} T cells are depicted. Data were pooled from two to three independent experiments, which included 4-9 mice per group and presented as mean + SD. Mann-Whitney test was used to test for statistical significance and significance was calculated by comparing values from untreated animals (0 dpi) and IAV-infected mice (2 to 21 dpi). Significant changes are indicated by an asterisk.

Supplementary Figure S3

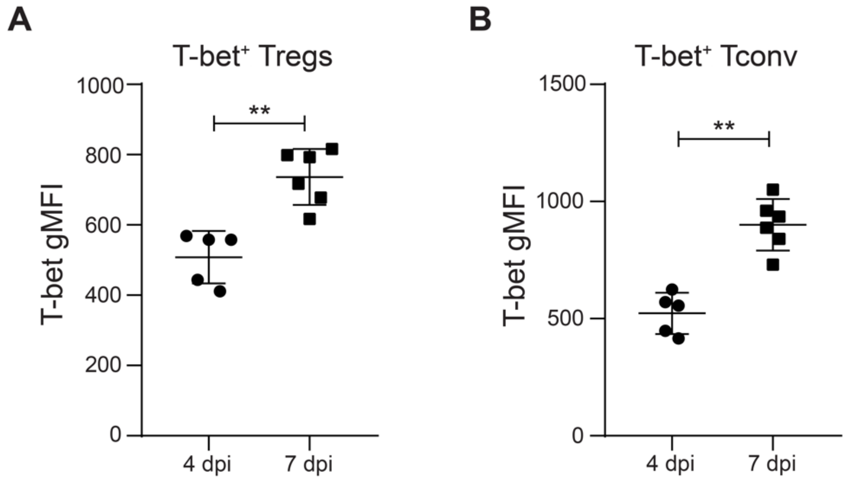

Figure S3: T-bet expression levels in T-bet$^+$ Tregs and Tconv from IAV-infected lungs. Foxp3^{hCD2} x Rag1^{GFP} reporter mice were infected with IAV and analyzed at indicated dpi. Lungs were collected and prepared, and single-cell suspensions were restimulated and subsequently analyzed by flow cytometry. Graphs summarize T-bet geometric mean fluorescence intensity (gMFI) of T-bet$^+$ Tregs (**a)** and T-bet$^+$ Tconv (**b**) at indicated time points. Data were pooled from two independent experiments, which included 4-5 mice per group, and presented as mean + SD. Mann-Whitney test was used to calculate statistical significance. Significant changes are indicated by an asterisk.

Supplementary Figure S4

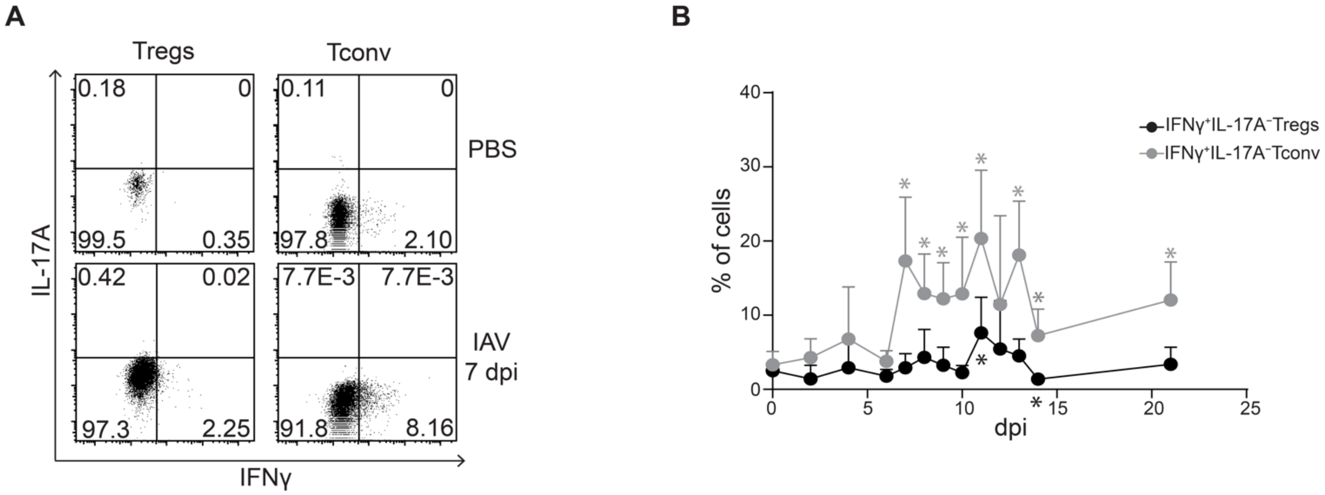

Figure S4: Cytokine expression in Tregs and Tconv from IAV-infected lungs. Foxp3^{hCD2} x Rag1^{GFP} reporter mice were infected with IAV and analyzed at indicated dpi. Lungs were collected and prepared, and single-cell suspensions were restimulated and subsequently analyzed by flow cytometry. (**a**) Representative dot plots depict IL-17A and IFN-γ expression in Tregs and Tconv from IAV-infected mice (7 dpi) and PBS-treated controls. Numbers indicate the frequencies in the corresponding quadrants. (**b**) Graphs summarize frequencies of IFN-γ^{+}IL-17A^{-} Tregs (black) and Tconv (grey) at indicated time points. Data were pooled from two independent experiments, which included 4-9 mice per group, and presented as mean + SD. Mann-Whitney test was used to calculate statistical significance by comparing values from PBS-treated animals (0 dpi) with values from IAV-infected mice (2 to 21 dpi). Significant changes are indicated by an asterisk.

Supplementary Figure S5

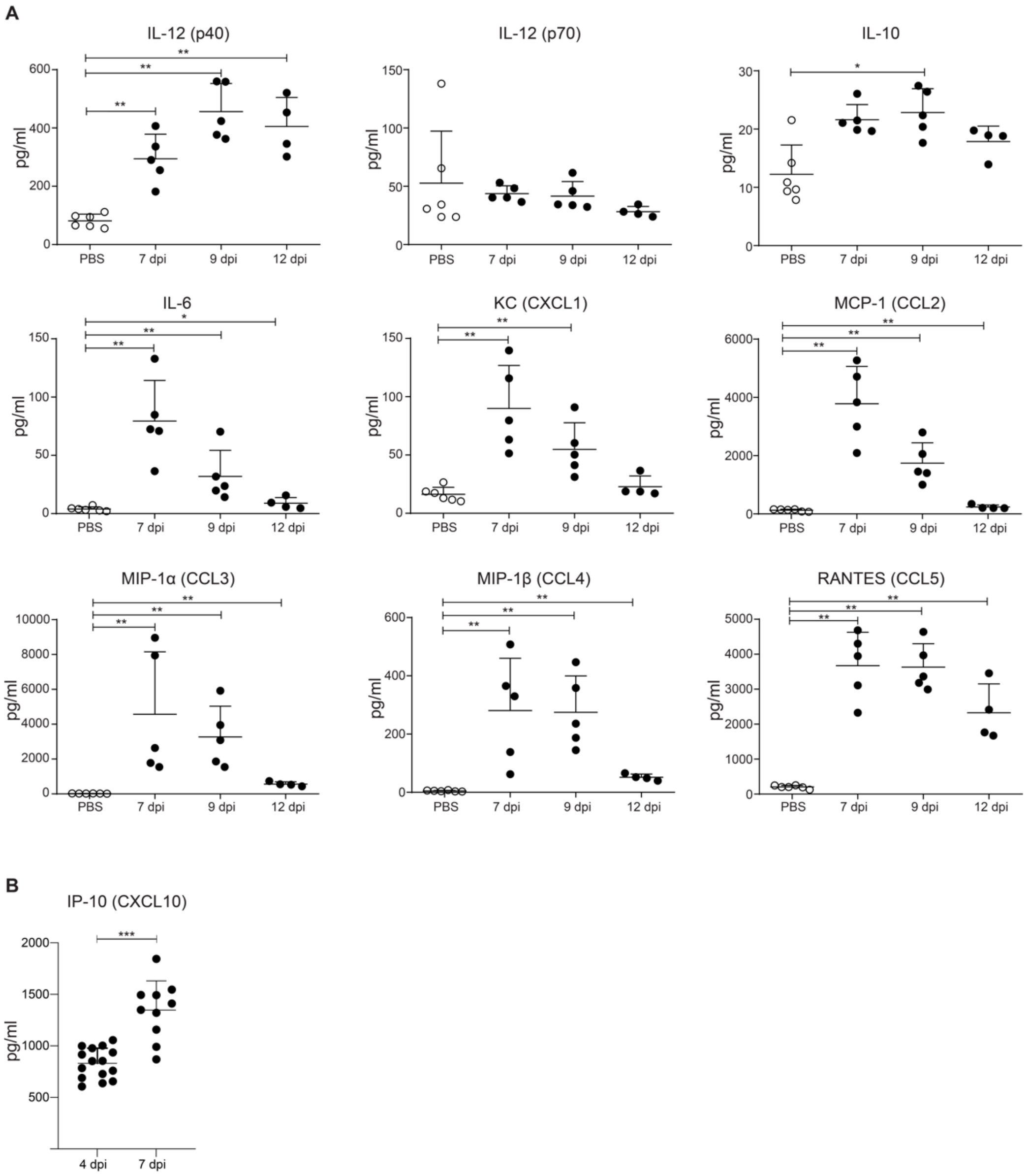

Figure S5: Cytokine/chemokine expression in IAV infected lungs. Foxp3^{hCD2} x Rag1^{GFP} reporter mice were infected with IAV and analyzed at indicated dpi. (A) Lungs were collected and prepared, homogenized, lysed and analyzed with a Bio-Plex Pro Mouse Cytokine Assay. (B) The concentration of the chemokine IP-10 (CXCL10) in the BALF was measured with a CXCL10/IP-10/CRG-2 DuoSet ELISA. Graphs summarize the indicated cytokine or chemokine concentration in PBS-treated (PBS, clear circles) or IAV-infected animals at indicated time points post infection (filled circles). Data were pooled from two to three independent experiments, which included 2 to 5 mice per group, and presented as mean + SD. Mann-Whitney test (A) was used to calculate statistical significance by comparing values from PBS-treated animals (0 dpi) with values from IAV-infected mice (7 to 12 dpi). A two-tailed, unpaired t test with Welch´s correction (B) was performed to identify statistical significance between the CXCL10 concentrations at 4 and 7 dpi. Significant changes are indicated by an asterisk.

Supplementary Figure S6

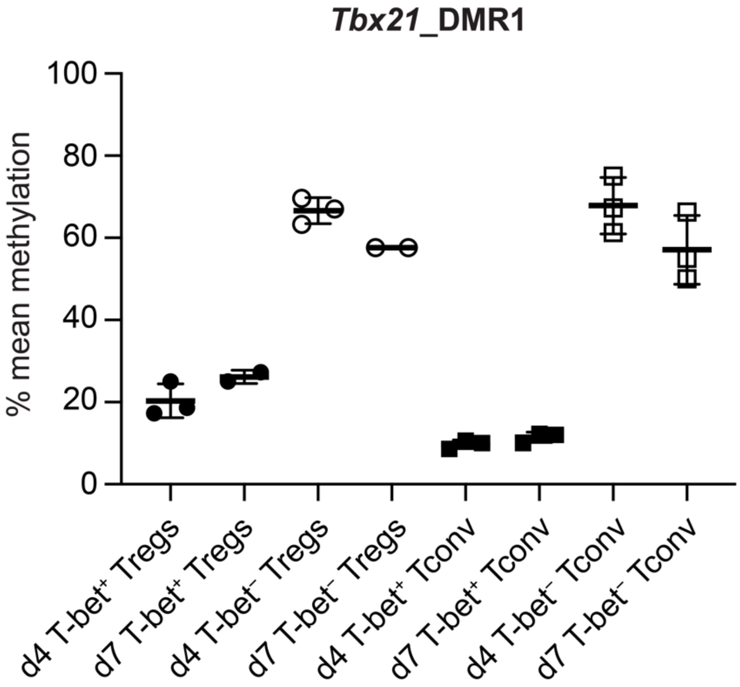

Figure S6: Methylation analysis of *Tbx21*_DMR1 in T-bet$^+$ and T-bet$^-$ Tregs and Tconv from IAV-infected lungs. Lungs from IAV-infected animals were collected and prepared at 4 or 7 dpi. T-bet$^+$ and T-bet$^-$ Treg as well as Tconv subsets were immediately sorted by flow cytometry (see Figure S1), and genomic DNA was prepared. The methylation status of *Tbx21*_DMR1 was determined by pyrosequencing. The graph shows the mean methylation of *Tbx21*_DMR1 for T-bet$^+$ Tregs (black filled circle), T-bet$^-$ Tregs (open circle), T-bet$^+$ Tconv (black filled square), or T-bet$^-$ Tconv (open square) populations. Each symbol represents data from cells isolated from 2-5 IAV-infected mice, and data were pooled from two to three independent experiments.

Supplementary Table S1: Summary of p-value calculations

Figure	Sample description	Comparison	p-value	p-value summary
1A	% $CD3^+$ cells	0 dpi vs 2 dpi	0.1709	ns
		0 dpi vs 4 dpi	0.1274	ns
		0 dpi vs 5 dpi	0.004	**
		0 dpi vs 6 dpi	0.0016	**
		0 dpi vs 7 dpi	0.0293	*
		0 dpi vs 8 dpi	0.0007	***
		0 dpi vs 9 dpi	<0.0001	****
		0 dpi vs 10 dpi	<0.0001	****
		0 dpi vs 11 dpi	0.0007	***
		0 dpi vs 12 dpi	0.0932	ns
		0 dpi vs 13 dpi	0.0653	ns
		0 dpi vs 14 dpi	0.0047	**
		0 dpi vs 21 dpi	0.3543	ns
1A	# $CD3^+$ cells	0 dpi vs 2 dpi	0.8329	ns
		0 dpi vs 4 dpi	0.0653	ns
		0 dpi vs 5 dpi	0.0727	ns
		0 dpi vs 6 dpi	0.5237	ns
		0 dpi vs 7 dpi	0.0007	***
		0 dpi vs 8 dpi	0.0007	***
		0 dpi vs 9 dpi	<0.0001	****
		0 dpi vs 10 dpi	<0.0001	****
		0 dpi vs 11 dpi	0.0007	***
		0 dpi vs 12 dpi	0.0653	ns
		0 dpi vs 13 dpi	0.0062	**
		0 dpi vs 14 dpi	0.0007	***
		0 dpi vs 21 dpi	0.1274	ns
1B	% $CD4^+$ cells	0 dpi vs 2 dpi	0.4351	ns
		0 dpi vs 4 dpi	0.0031	**
		0 dpi vs 5 dpi	0.0061	**
		0 dpi vs 6 dpi	0.0062	**
		0 dpi vs 7 dpi	0.0013	**
		0 dpi vs 8 dpi	0.0053	**
		0 dpi vs 9 dpi	<0.0001	****
		0 dpi vs 10 dpi	<0.0001	****
		0 dpi vs 11 dpi	0.0007	***
		0 dpi vs 12 dpi	0.007	**
		0 dpi vs 13 dpi	0.0016	**
		0 dpi vs 14 dpi	0.0013	**
		0 dpi vs 21 dpi	0.0016	**

1B	% $CD8^+$ cells	0 dpi vs 2 dpi	0.6954	ns
		0 dpi vs 4 dpi	0.0109	*
		0 dpi vs 5 dpi	0.0687	ns
		0 dpi vs 6 dpi	0.0109	*
		0 dpi vs 7 dpi	0.002	**
		0 dpi vs 8 dpi	0.0013	**
		0 dpi vs 9 dpi	<0.0001	****
		0 dpi vs 10 dpi	<0.0001	****
		0 dpi vs 11 dpi	0.0007	***
		0 dpi vs 12 dpi	0.0109	*
		0 dpi vs 13 dpi	0.0016	**
		0 dpi vs 14 dpi	0.0013	**
		0 dpi vs 21 dpi	0.0016	**
1B	# $CD4^+$ cells	0 dpi vs 2 dpi	0.8329	ns
		0 dpi vs 4 dpi	0.2844	ns
		0 dpi vs 5 dpi	0.4606	ns
		0 dpi vs 6 dpi	0.6216	ns
		0 dpi vs 7 dpi	0.0007	***
		0 dpi vs 8 dpi	0.0007	***
		0 dpi vs 9 dpi	<0.0001	****
		0 dpi vs 10 dpi	<0.0001	****
		0 dpi vs 11 dpi	0.0007	***
		0 dpi vs 12 dpi	0.0482	*
		0 dpi vs 13 dpi	0.0124	*
		0 dpi vs 14 dpi	0.0007	***
		0 dpi vs 21 dpi	0.7242	ns
1B	# $CD8^+$ cells	0 dpi vs 2 dpi	>0.9999	ns
		0 dpi vs 4 dpi	0.0186	*
		0 dpi vs 5 dpi	0.0727	ns
		0 dpi vs 6 dpi	0.5237	ns
		0 dpi vs 7 dpi	0.0007	***
		0 dpi vs 8 dpi	0.0007	***
		0 dpi vs 9 dpi	<0.0001	****
		0 dpi vs 10 dpi	<0.0001	****
		0 dpi vs 11 dpi	0.0007	***
		0 dpi vs 12 dpi	0.0653	ns
		0 dpi vs 13 dpi	0.0016	**
		0 dpi vs 14 dpi	0.0007	***
		0 dpi vs 21 dpi	0.0653	ns
1C	% $CD3^+$ cells	0 dpi vs 4 dpi	0.0193	*
		0 dpi vs 5 dpi	0.008	**
		0 dpi vs 6 dpi	0.0992	ns
		0 dpi vs 7 dpi	0.003	**
		0 dpi vs 8 dpi	0.0002	***

		0 dpi vs 9 dpi	0.0003	***
		0 dpi vs 10 dpi	0.011	*
		0 dpi vs 11 dpi	0.0071	**
		0 dpi vs 12 dpi	0.0013	**
		0 dpi vs 13 dpi	0.0013	**
		0 dpi vs 14 dpi	0.0127	*
		0 dpi vs 21 dpi	0.0027	**
1C	# $CD3^+$ cells	0 dpi vs 4 dpi	0.8591	ns
		0 dpi vs 5 dpi	0.5395	ns
		0 dpi vs 6 dpi	0.2544	ns
		0 dpi vs 7 dpi	0.0934	ns
		0 dpi vs 8 dpi	0.4923	ns
		0 dpi vs 9 dpi	0.0172	*
		0 dpi vs 10 dpi	0.9578	ns
		0 dpi vs 11 dpi	0.5362	ns
		0 dpi vs 12 dpi	0.6787	ns
		0 dpi vs 13 dpi	0.7679	ns
		0 dpi vs 14 dpi	0.3097	ns
		0 dpi vs 21 dpi	0.8591	ns
1D	% $CD4^+$ cells	0 dpi vs 4 dpi	0.013	*
		0 dpi vs 5 dpi	0.002	**
		0 dpi vs 6 dpi	>0.9999	ns
		0 dpi vs 7 dpi	0.2098	ns
		0 dpi vs 8 dpi	0.6917	ns
		0 dpi vs 9 dpi	0.2337	ns
		0 dpi vs 10 dpi	0.1105	ns
		0 dpi vs 11 dpi	0.7191	ns
		0 dpi vs 12 dpi	0.4938	ns
		0 dpi vs 13 dpi	0.004	**
		0 dpi vs 14 dpi	0.1555	ns
		0 dpi vs 21 dpi	0.3886	ns
1D	% $CD8^+$ cells	0 dpi vs 4 dpi	0.022	*
		0 dpi vs 5 dpi	0.002	**
		0 dpi vs 6 dpi	0.5325	ns
		0 dpi vs 7 dpi	0.3676	ns
		0 dpi vs 8 dpi	0.3822	ns
		0 dpi vs 9 dpi	0.003	**
		0 dpi vs 10 dpi	0.002	**
		0 dpi vs 11 dpi	0.9064	ns
		0 dpi vs 12 dpi	0.5135	ns
		0 dpi vs 13 dpi	0.0027	**
		0 dpi vs 14 dpi	0.0753	ns
		0 dpi vs 21 dpi	0.028	*

1D	# $CD4^+$ cells	0 dpi vs 4 dpi	0.8392	ns
		0 dpi vs 5 dpi	0.3736	ns
		0 dpi vs 6 dpi	0.2544	ns
		0 dpi vs 7 dpi	0.1179	ns
		0 dpi vs 8 dpi	0.4923	ns
		0 dpi vs 9 dpi	0.0172	*
		0 dpi vs 10 dpi	0.7128	ns
		0 dpi vs 11 dpi	0.6009	ns
		0 dpi vs 12 dpi	0.7679	ns
		0 dpi vs 13 dpi	0.7333	ns
		0 dpi vs 14 dpi	0.3097	ns
		0 dpi vs 21 dpi	0.8591	ns
1D	# $CD8^+$ cells	0 dpi vs 4 dpi	0.7333	ns
		0 dpi vs 5 dpi	0.5395	ns
		0 dpi vs 6 dpi	0.3097	ns
		0 dpi vs 7 dpi	0.0727	ns
		0 dpi vs 8 dpi	0.3676	ns
		0 dpi vs 9 dpi	0.0133	*
		0 dpi vs 10 dpi	>0.9999	ns
		0 dpi vs 11 dpi	0.5362	ns
		0 dpi vs 12 dpi	0.7679	ns
		0 dpi vs 13 dpi	0.8591	ns
		0 dpi vs 14 dpi	0.2544	ns
		0 dpi vs 21 dpi	0.8591	ns
2B	% lung Tregs	0 dpi vs 2 dpi	0.0451	*
		0 dpi vs 4 dpi	0.0016	**
		0 dpi vs 5 dpi	0.004	**
		0 dpi vs 6 dpi	0.0016	**
		0 dpi vs 7 dpi	0.0007	***
		0 dpi vs 8 dpi	0.02	*
		0 dpi vs 9 dpi	0.0036	**
		0 dpi vs 10 dpi	0.0037	**
		0 dpi vs 11 dpi	0.0013	**
		0 dpi vs 12 dpi	0.0186	*
		0 dpi vs 13 dpi	0.0062	**
		0 dpi vs 14 dpi	0.0047	**
		0 dpi vs 21 dpi	0.0016	**
2B	# lung Tregs	0 dpi vs 2 dpi	0.2844	ns
		0 dpi vs 4 dpi	0.0186	*
		0 dpi vs 5 dpi	0.0081	**
		0 dpi vs 6 dpi	0.5237	ns
		0 dpi vs 7 dpi	0.0007	***
		0 dpi vs 8 dpi	0.0007	***

		0 dpi vs 9 dpi	<0.0001	****
		0 dpi vs 10 dpi	<0.0001	****
		0 dpi vs 11 dpi	0.0007	***
		0 dpi vs 12 dpi	0.0202	*
		0 dpi vs 13 dpi	0.0062	**
		0 dpi vs 14 dpi	0.0007	***
		0 dpi vs 21 dpi	0.1274	ns
2B	# lung Tconv	0 dpi vs 2 dpi	0.8329	ns
		0 dpi vs 4 dpi	0.2844	ns
		0 dpi vs 5 dpi	0.8081	ns
		0 dpi vs 6 dpi	0.6216	ns
		0 dpi vs 7 dpi	0.0007	***
		0 dpi vs 8 dpi	0.0007	***
		0 dpi vs 9 dpi	<0.0001	****
		0 dpi vs 10 dpi	<0.0001	****
		0 dpi vs 11 dpi	0.0007	***
		0 dpi vs 12 dpi	0.0653	ns
		0 dpi vs 13 dpi	0.0186	*
		0 dpi vs 14 dpi	0.0007	***
		0 dpi vs 21 dpi	0.8329	ns
2D	% dLN Tregs	0 dpi vs 4 dpi	0.965	ns
		0 dpi vs 5 dpi	0.2498	ns
		0 dpi vs 6 dpi	0.1722	ns
		0 dpi vs 7 dpi	0.012	*
		0 dpi vs 8 dpi	0.2752	ns
		0 dpi vs 9 dpi	0.3257	ns
		0 dpi vs 10 dpi	0.2515	ns
		0 dpi vs 11 dpi	0.2282	ns
		0 dpi vs 12 dpi	0.0693	ns
		0 dpi vs 13 dpi	0.9764	ns
		0 dpi vs 14 dpi	0.6787	ns
		0 dpi vs 21 dpi	0.371	ns
2D	# dLN Tregs	0 dpi vs 4 dpi	>0.9999	ns
		0 dpi vs 5 dpi	0.6828	ns
		0 dpi vs 6 dpi	0.2222	ns
		0 dpi vs 7 dpi	0.1079	ns
		0 dpi vs 8 dpi	0.7546	ns
		0 dpi vs 9 dpi	0.0592	ns
		0 dpi vs 10 dpi	0.662	ns
		0 dpi vs 11 dpi	0.7789	ns
		0 dpi vs 12 dpi	0.9433	ns
		0 dpi vs 13 dpi	0.3543	ns
		0 dpi vs 14 dpi	0.5237	ns
		0 dpi vs 21 dpi	0.7242	ns

2D	# dLN Tconv	0 dpi vs 4 dpi	0.7242	ns
		0 dpi vs 5 dpi	0.6828	ns
		0 dpi vs 6 dpi	0.1274	ns
		0 dpi vs 7 dpi	0.345	ns
		0 dpi vs 8 dpi	0.9497	ns
		0 dpi vs 9 dpi	0.0592	ns
		0 dpi vs 10 dpi	0.2824	ns
		0 dpi vs 11 dpi	0.7789	ns
		0 dpi vs 12 dpi	0.9433	ns
		0 dpi vs 13 dpi	0.1709	ns
		0 dpi vs 14 dpi	0.5237	ns
		0 dpi vs 21 dpi	0.7242	ns
3A	% T-bet^{+}RORγt^{-} Tregs	0 dpi vs 2 dpi	0.2222	ns
		0 dpi vs 4 dpi	0.3543	ns
		0 dpi vs 6 dpi	0.1274	ns
		0 dpi vs 7 dpi	0.0007	***
		0 dpi vs 8 dpi	0.0007	***
		0 dpi vs 9 dpi	0.0745	ns
		0 dpi vs 10 dpi	0.1419	ns
		0 dpi vs 11 dpi	0.0093	**
		0 dpi vs 12 dpi	0.0653	ns
		0 dpi vs 13 dpi	0.3543	ns
		0 dpi vs 14 dpi	0.8283	ns
		0 dpi vs 21 dpi	0.7242	ns
3A	% T-bet^{-}RORγt^{+} Tregs	0 dpi vs 2 dpi	0.0435	*
		0 dpi vs 4 dpi	0.2999	ns
		0 dpi vs 6 dpi	0.2797	ns
		0 dpi vs 7 dpi	0.0196	*
		0 dpi vs 8 dpi	0.0196	*
		0 dpi vs 9 dpi	0.0109	*
		0 dpi vs 10 dpi	0.0123	*
		0 dpi vs 11 dpi	0.0389	*
		0 dpi vs 12 dpi	0.0179	*
		0 dpi vs 13 dpi	0.0622	ns
		0 dpi vs 14 dpi	0.0014	**
		0 dpi vs 21 dpi	0.5221	ns
3B	% T-bet^{+}RORγt^{-} Tconv	0 dpi vs 2 dpi	0.2222	ns
		0 dpi vs 4 dpi	0.5237	ns
		0 dpi vs 6 dpi	0.4351	ns
		0 dpi vs 7 dpi	0.0007	***
		0 dpi vs 8 dpi	0.0007	***
		0 dpi vs 9 dpi	0.2359	ns

		0 dpi vs 10 dpi	0.2824	ns
		0 dpi vs 11 dpi	0.0037	**
		0 dpi vs 12 dpi	0.3543	ns
		0 dpi vs 13 dpi	0.5237	ns
		0 dpi vs 14 dpi	0.5148	ns
		0 dpi vs 21 dpi	0.021	*
3B	% T-bet^{-}RORγt^{+} Tconv	0 dpi vs 2 dpi	0.2844	ns
		0 dpi vs 4 dpi	0.9433	ns
		0 dpi vs 6 dpi	0.0653	ns
		0 dpi vs 7 dpi	0.0813	ns
		0 dpi vs 8 dpi	0.02	*
		0 dpi vs 9 dpi	0.3213	ns
		0 dpi vs 10 dpi	0.5728	ns
		0 dpi vs 11 dpi	0.7789	ns
		0 dpi vs 12 dpi	0.0062	**
		0 dpi vs 13 dpi	0.1274	ns
		0 dpi vs 14 dpi	<0.0001	****
		0 dpi vs 21 dpi	0.6216	ns
4B	methylation *Tbx21*_DMR1 Tregs	T-bet^{+} vs T-bet^{-}	0.2677	ns
	methylation *Tbx21*_DMR1 Tconv	T-bet^{+} vs T-bet^{-}	0.0017	**
	methylation *Tbx21*_DMR2 Tregs	T-bet^{+} vs T-bet^{-}	0.0025	**
	methylation *Tbx21*_DMR2 Tconv	T-bet^{+} vs T-bet^{-}	0.0006	***
	methylation TSDR Tregs	T-bet^{+} vs T-bet^{-}	0.0571	ns
	methylation TSDR Tconv	T-bet^{+} vs T-bet^{-}	0.0146	*
S2A	% CD3^{+} cells	0 dpi vs 2 dpi	0.0317	*
		0 dpi vs 4 dpi	0.1508	ns
		0 dpi vs 5 dpi	0.2857	ns
		0 dpi vs 6 dpi	0.2222	ns
		0 dpi vs 7 dpi	0.4286	ns
		0 dpi vs 8 dpi	0.0173	*
		0 dpi vs 9 dpi	0.007	**
		0 dpi vs 10 dpi	0.007	**
		0 dpi vs 11 dpi	0.0025	**
		0 dpi vs 12 dpi	0.0159	*
		0 dpi vs 13 dpi	0.0079	**
		0 dpi vs 14 dpi	0.0043	**
		0 dpi vs 21 dpi	0.0952	ns
S2B	% CD4^{+} cells	0 dpi vs 2 dpi	0.1508	ns
		0 dpi vs 4 dpi	0.5952	ns
		0 dpi vs 5 dpi	0.0635	ns
		0 dpi vs 6 dpi	0.0078	**
		0 dpi vs 7 dpi	0.1623	ns
		0 dpi vs 8 dpi	0.0714	ns

		0 dpi vs 9 dpi	0.0599	ns
		0 dpi vs 10 dpi	0.1563	ns
		0 dpi vs 11 dpi	0.1351	ns
		0 dpi vs 12 dpi	0.1508	ns
		0 dpi vs 13 dpi	0.1508	ns
		0 dpi vs 14 dpi	0.1255	ns
		0 dpi vs 21 dpi	0.0556	ns
S2B	% $CD8^+$ cells	0 dpi vs 2 dpi	0.0952	ns
		0 dpi vs 4 dpi	0.3095	ns
		0 dpi vs 5 dpi	0.0317	*
		0 dpi vs 6 dpi	0.0016	**
		0 dpi vs 7 dpi	0.0823	ns
		0 dpi vs 8 dpi	0.0519	ns
		0 dpi vs 9 dpi	0.1469	ns
		0 dpi vs 10 dpi	0.0899	ns
		0 dpi vs 11 dpi	0.202	ns
		0 dpi vs 12 dpi	0.0556	ns
		0 dpi vs 13 dpi	0.0952	ns
		0 dpi vs 14 dpi	0.0303	*
		0 dpi vs 21 dpi	0.0079	**
S2C	% Tregs	0 dpi vs 2 dpi	0.0079	**
		0 dpi vs 4 dpi	0.5476	ns
		0 dpi vs 5 dpi	0.7302	ns
		0 dpi vs 6 dpi	0.6216	ns
		0 dpi vs 7 dpi	0.9675	ns
		0 dpi vs 8 dpi	0.2684	ns
		0 dpi vs 9 dpi	0.6993	ns
		0 dpi vs 10 dpi	0.8981	ns
		0 dpi vs 11 dpi	0.0316	*
		0 dpi vs 12 dpi	0.0952	ns
		0 dpi vs 13 dpi	0.5476	ns
		0 dpi vs 14 dpi	0.9307	ns
		0 dpi vs 21 dpi	0.8413	ns
S3B	% $IFN\gamma^+IL\text{-}17A^-$ Tregs	0 dpi vs 2 dpi	0.3349	ns
		0 dpi vs 4 dpi	0.2222	ns
		0 dpi vs 6 dpi	0.3543	ns
		0 dpi vs 8 dpi	0.9497	ns
		0 dpi vs 7 dpi	0.8518	ns
		0 dpi vs 9 dpi	>0.9999	ns
		0 dpi vs 10 dpi	0.9497	ns
		0 dpi vs 11 dpi	0.021	*
		0 dpi vs 12 dpi	0.7242	ns
		0 dpi vs 13 dpi	0.1018	ns
		0 dpi vs 14 dpi	0.0426	*

		0 dpi vs 21 dpi	0.6488	ns
S3B	% IFNγ^{+}IL-17A^{-} Tconv	0 dpi vs 2 dpi	0.4351	ns
		0 dpi vs 4 dpi	0.4351	ns
		0 dpi vs 6 dpi	0.4351	ns
		0 dpi vs 8 dpi	0.0007	***
		0 dpi vs 7 dpi	0.0027	**
		0 dpi vs 9 dpi	0.0016	**
		0 dpi vs 10 dpi	0.0007	***
		0 dpi vs 11 dpi	0.0003	***
		0 dpi vs 12 dpi	0.1274	ns
		0 dpi vs 13 dpi	0.0016	**
		0 dpi vs 14 dpi	0.0293	*
		0 dpi vs 21 dpi	0.0016	**
